# Predictors of immunological non-response despite virological suppression in people living with HIV on antiretroviral therapy: A systematic review and meta-analysis

**DOI:** 10.1371/journal.pone.0358793

**Published:** 2026-10-12

**Authors:** Brian Eka Rachman, Yehuda Tri Nugroho Supranoto, Fitri Revita Nurannisa, Siti Mei Saroh, Siti Qamariyah Khairunisa, Citrawati Dyah Kencono Wungu, Dian Paramita Kartikasari, Nasronudin Nasronudin, Masanori Kameoka, Muhammad Miftahussurur, Retno Pudji Rahayu, Afif Nurul Hidayati

**Affiliations:** 1 Doctoral Program of Medical Science, Faculty of Medicine, Universitas Airlangga, Surabaya, Indonesia; 2 Division of Tropical Medicine and Infectious Diseases, Department of Internal Medicine, Faculty of Medicine, Universitas Airlangga, Surabaya, Indonesia; 3 Master of Medical Sciences in Clinical Investigation, Harvard Medical School, Boston, Massachusetts, United States of America; 4 Master of Public Health Program, Faculty of Medicine, Public Health, and Nursing, Universitas Gadjah Mada, Yogyakarta, Indonesia; 5 Center for Tropical Medicine, Faculty of Medicine, Universitas Gadjah Mada, Yogyakarta, Indonesia; 6 Institute of Tropical Disease, Universitas Airlangga, Surabaya, Indonesia; 7 Department of Physiology and Medical Biochemistry, Faculty of Medicine, Universitas Airlangga, Surabaya, Indonesia; 8 Department of Cardiology and Vascular Medicine, Faculty of Medicine, Universitas Airlangga, Surabaya, Indonesia; 9 Universitas Airlangga Hospital, Surabaya, Indonesia; 10 Division of Global and Environmental Health Sciences, Department of Future Social Medicine, Kobe University Graduate School of Medicine, Kobe, Japan; 11 Division of Gastroenterohepatology, Department of Internal Medicine, Faculty of Medicine, Dr. Soetomo Teaching Hospital, Universitas Airlangga, Surabaya, Indonesia; 12 Helicobacter pylori and Microbiota Study Group, Institute Tropical Disease, Universitas Airlangga, Surabaya, Indonesia; 13 Department of Oral and Maxillofacial Pathology, Faculty of Dentistry, Universitas Airlangga, Surabaya, Indonesia; 14 Department of Dermatology Venereology and Aesthetics, Faculty of Medicine, Universitas Airlangga, Surabaya, Indonesia; University of Verona, ITALY

## Abstract

Immunological Nonresponse (INR) in people living with HIV (PLWH) is characterized by inadequate CD4^+^ T-cell recovery despite effective viral suppression through antiretroviral therapy (ART). This systematic review and meta-analysis identify key clinical, demographic, and molecular predictors of INR to optimize immune recovery strategies. A comprehensive literature search was conducted in accordance with PRISMA 2020 guidelines, yielding 59 eligible studies, with a total of 19,511 individuals with INR. Study quality was assessed using the Newcastle-Ottawa Scale (NOS), and pooled effect estimates were calculated as odds ratio (OR), mean difference (MD), or standardized mean difference (SMD). Significant predictors of INR included male sex [OR 1.33 (1.12–1.58)], older age [MD 2.61 (1.42–3.81)], intravenous drug use [OR 1.67 (1.15–2.42)], hepatitis B coinfection [OR 1.30 (1.08–1.57)], hepatitis C coinfection [OR 1.85 (1.50–2.28)], tuberculosis [OR 1.77 (1.41–2.21)], advanced HIV stage [OR 2.66 (2.34–3.02)], and lower baseline and nadir CD4^+^ counts [SMD −3.82 (−4.94-(−2.70)), SMD −1.69 (−2.69-(−0.68))], respectively. Additionally, 57 biomarkers were identified as potential predictors of INR, implicating several immunological pathways. These findings underscore the multifactorial nature of INR and the need for personalized approaches among ART-treated individuals.

## Introduction

The introduction of antiretroviral therapy (ART) has dramatically transformed the outlook for people living with HIV (PLWH), enabling sustained viral suppression and reducing HIV-related morbidity and mortality. However, a considerable proportion, approximately 10–40% of individuals, do not experience sufficient recovery of CD4⁺ T-cell counts despite achieving virological suppression, a condition referred to as immunological non-response (INR) [1–3]. There is currently no universally accepted definition of INR. Across studies, INR has been operationalized using different absolute CD4⁺ T-cell thresholds, commonly <200, < 350, or <500 cells/µL, failure to achieve a predefined increase in CD4⁺ T-cell count from baseline, and varying durations of ART or virological suppression [2]. This lack of a standardized definition may affect the classification and reported prevalence of INR and contribute to heterogeneity in the identification of its associated predictors, thereby complicating comparisons across studies and highlighting the need for systematic evidence synthesis. Importantly, INR has been linked to a 61% higher risk of developing serious non-AIDS events (SNAEs) such as cardiovascular disease, malignancies, renal impairment, neurocognitive disorders, and severe infections over long-term follow-up [3].

Several studies have investigated demographic, clinical, genetic, and molecular factors as potential contributors to the risk of INR. Among demographic variables, increasing age has consistently been linked to suboptimal immune recovery, likely due to immunosenescence and a diminished capacity for T-cell regeneration [4]. Male sex has similarly been associated with a greater risk of INR, which may be influenced by hormonal differences and sex-based immunological variation [5,6]. In addition, the mode of HIV transmission appears to play a role, with people who inject drugs (PWID) exhibiting a higher prevalence of INR, possibly driven by persistent immune activation and co-existing infections [7,8].

Clinical factors have also been implicated in INR. Chronic co-infections, particularly with hepatitis B, hepatitis C, and tuberculosis, can sustain systemic inflammation and immune activation, potentially impairing CD4⁺ T-cell reconstitution [9,10]. Furthermore, low body mass index (BMI), which may reflect underlying malnutrition or metabolic dysfunction, has also been reported as a risk factor for poor immune recovery [11].

Genetic and molecular factors offer further insight into the complex pathogenesis of INR. Variants in genes related to the Human Leukocyte Antigen (HLA) system and Killer-cell Immunoglobulin-like Receptors (KIR) have been associated with immune modulation and response to ART [12–14]. Likewise, polymorphisms in chemokine receptors, which regulate immune cell trafficking and inflammatory signaling, may also influence the degree of immune reconstitution [15–17]. A range of molecular indicators, including inflammatory cytokines, metabolic regulators, and immune activation markers, underscore the intricate crosstalk between inflammation, metabolic dysfunction, and immune restoration [16,18,19].

Despite substantial research, evidence linking INR to its potential risk factors remains inconclusive. While some studies have reported strong associations between age and INR [20,21], others have found little to no significant relationship [1]. A similar pattern of conflicting results is observed in studies examining the roles of co-infections and genetic markers [15,22–26], which may be attributable to differences in study populations, methodological approaches, or how INR is defined across investigations. Moreover, most studies have examined INR from a single perspective, focusing solely on demographic, clinical, or molecular variables, without providing an integrated analysis of their relative contributions. This lack of consistency in study designs and inclusion criteria has further added to the heterogeneity of findings, posing challenges for drawing clear, evidence-based conclusions.

To bridge existing knowledge gaps, this study presents what, to our knowledge, is the first systematic review and meta-analysis that comprehensively evaluates demographic, clinical, genetic, and molecular predictors of INR. By synthesizing findings from a wide range of studies, this work aims to quantify the relative contribution of each factor through pooled effect estimates and to clarify the inconsistencies observed in the existing literature. Through this integrative approach, the study seeks to enhance the current understanding of INR pathogenesis and inform the development of more targeted and individualized strategies to improve immune recovery in ART-treated individuals.

## Methods

This systematic review and meta-analysis was conducted and reported in accordance with the Preferred Reporting Items for Systematic Reviews and Meta-Analyses 2020 (PRISMA 2020) statement [27] and the recommendations of the Cochrane Handbook for Systematic Reviews of Interventions [28] (S1 File). The protocol was prospectively registered in the International Prospective Register of Systematic Reviews (PROSPERO) [29] under registration number CRD42021258880.

### Eligibility criteria

Studies were included if they met the following criteria: (1) studies reporting on people living with HIV (PLWH) who were classified as having immunological non-response (INR) during antiretroviral therapy (ART), using operational definitions consistent with the spectrum of criteria summarized by Rb-Silva et al. [2]. Rather than applying a single CD4⁺ T-cell cutoff, eligible definitions could be based on absolute CD4⁺ T-cell thresholds, changes in CD4⁺ T-cell counts from baseline, or combinations of these measures assessed at defined time points during ART and/or virological suppression; (2) observational study designs, including cohort, case-control, or cross-sectional studies; (3) studies including a comparator group with adequate immunological response (IR) and reporting differences in at least one of the following domains: demographic characteristics, clinical factors, genetic variants, or circulating molecular biomarkers; (4) studies providing quantitative data sufficient for data extraction and, where applicable, estimation of effect measures; (5) studies conducted in human participants; and (6) studies published in English. Studies were excluded if they were: (1) review articles, systematic reviews, meta-analyses, clinical trials, case reports, or case series; (2) duplicate publications; (3) studies with insufficient quantitative data to extract at least one eligible predictor–INR comparison. Data were considered incomplete when the information required for the relevant analysis could neither be extracted nor calculated from the reported results, such as the absence of group-specific counts for dichotomous variables or insufficient summary statistics for continuous variables. Studies providing usable quantitative data for at least one eligible predictor were retained even when data for other variables were not reported; (4) conference abstracts, letters to the editor, or abstract-only publications; or (5) studies without a clearly stated definition or classification of immunological response status.

For consistency across studies, the terminology for immunological response status was standardized throughout this review. Groups representing inadequate immunological recovery were categorized as having immunological non-response (INR), whereas the corresponding adequate-response groups were categorized as having immunological response (IR), irrespective of the terminology used in the original studies. The study-specific operational definitions, including absolute CD4⁺ T-cell thresholds, changes from baseline, and assessment time points, were retained as originally reported and are presented in Table 1.

**Table 1:** Characteristics of included studies.

Author, Year	Country	Study Design	Sample Size INR (%)	Age (Mean ± SD)	INR Definition	NOS
**Shi *et al.,*** 2025 [30]	China	Prospective cohort	20 (40.00)	4,1 ± 10,37	INR was defined as failure to achieve an increase in CD4⁺ T-cell count of ≥30% or ≥100 cells/μL from baseline after one year of antiretroviral therapy (ART).	8
**Sembiring *et al.*,** 2024 [31]	Indonesia	Prospective Cohort	55 (44.72)	37.51 ± 9.9	CD4 count <350 cells/mm and viral load <40 copies/mL after ARV therapy for at least one year.	6
**Guedes *et al.*,** 2023 [32]	Brazil	Prospective Cohort	13 (29.55)	41.8 ± 3	CD4^+^ T cell count <500 cells/µL and maintained CD4^+^ levels below this threshold after 18 months of ART despite achieving prolonged viral suppression (plasma viral load <40 copies/mL).	7
**Pereira *et al.*,** 2022 [12]	Brazil	Prospective Cohort	136 (50.37)	NR	CD4^+^ < 500 cells/μL after starting ART over 24 months.	9
**Merlini *et al.*,** 2021 [33]	Italy	Prospective Cohort	39 (31.0)	NR	People living with HIV (PLWH) on suppressive combination antiretroviral therapy (cART) fail to achieve significant CD4^+^ T-cell recovery despite effective viral suppression.	8
**Bahemana *et al.*,** 2020 [34]	Multi-countries	Prospective Cohort	Not clear	NR	Lack of significant increase in CD4 counts and lack of viral suppression within specific time frames, notably by comparing changes over periods up to 42 months post ART initiation.	8
**Carvalho-Silva *et al.*,** 2020 [17]	Brazil	Prospective Cohort	126 (50.81)	33.33 ± 7.5	Patients that gained less than 200 CD4^+^ T-cells/μL during the first year of ART, when compared with pretreatment count.	5
**Handoko *et al.*,** 2020 [35]	Thailand	Prospective Cohort	11 (4.23)	24.33 ± 8.48	• CD4^+^ < 350 cells/mm^3^ after a long duration of ART described as suboptimal immune recovery. • 350 <= CD4^+^ < 500 cells/mm^3^ after a long duration of ART described as intermediate immune recovery.	9
**Boatman *et al.*,** 2019 [36]	Multi-countries	Prospective Cohort	748 (39.70)	NR	CD4^+^ T cell count increased by less than 50 cells/mm^3^ from baseline after 8 months of initiating ART, despite having an HIV RNA level of < 200 copies/mL at 8 months.	9
**Greenblatt *et al.*,** 2019 [37]	US	Prospective Cohort	42 (46.67)	45.5 ± 8.2	Poor CD4 recovery, specifically those with the poorest gains in CD4 counts for at least one year while on virologically-suppressive cART.	8
**Li *et al.*,** 2019 [38]	China	Prospective Cohort	750 (48.00)	43.03 ± 10.42	Failing to achieve an increase in CD4^+^ T lymphocytes above 500 cells/μL during the follow-up after initiating Highly Active Antiretroviral Therapy (HAART) for 2 years.	9
**Rb-Silva *et al.*,** 2019 [39]	Portugal	Prospective Cohort	19 (57.58)	45.3 ± 10.8	Patients with virologic suppression (undetectable plasma HIV RNA) but persistently low CD4^+^ T cell counts, specifically having consistent CD4^+^ T cell counts below 500 cells/µL after at least 36 months of follow-up.	8
**Jiang *et al.*,** 2018 [40]	US	Prospective Cohort	Not clear	NR	Failure to recover peripheral CD4^+^ T cell counts despite being on long-term viral suppression (exact thresholds were not specified).	5
**Moore *et al.*,** 2007 [41]	US	Prospective Cohort	Not clear	NR	Patients who did not experience an increase in CD4^+^ cell count as expected after treatment with HAART (<500 cells/mL).	8
**Kaufmann *et al.*,** 2005 [42]	Switzerland	Prospective Cohort	105 (35.84)	40 ± 9.7	CD4 T cell counts < 500 cells/mL at 5 years of treatment.	6
**Nicastri *et al.*,** 2005 [43]	Italy	Prospective Cohort	585 (42.83)	35.1 ± 5.28	No increase in CD4 cell count with respect to the pre-HAART value of more than 100 cells/ml after 12 months.	6
**Rachman *et al*.,** 2025 [44]	Indonesia	Retrospective Cohort	207 (54.19)	34 ± 1,83	INR1: Failure to increase CD4+ T-cell count by ≥100 cells/mm³ from baseline after 2 years of ART. INR2: Failure to reach an absolute CD4+ T-cell count ≥350 cells/mm³ after 2 years of ART. INR3: Meeting either INR1 or INR2 criteria. INR4: Meeting both INR1 and INR2 criteria (most stringent). Patients who meet none of these criteria are classified as having immunological response (IR).	9
**Sun *et al*.,** 2025 [45]	China	Retrospective Cohort	9962 (88.26)	35 ± 2,83	INR is defined as having a total CD4+ T cell count of less than 350 cells/µL at 4 years after ART initiation.	9
**Tian *et al.*,** 2025 [46]	China	Retrospective Cohort	32 (45.71)	NR	Patients with absolute CD4^+^ T-cell count <200 cells/μL after 2 years of receiving complete viral-suppression ART.	7
**Vos *et al.*,** 2024 [47]	Netherlands	Retrospective Cohort	62 (4.82)	55.17 ± 12.14	CD4^+^ T cell count < 350 cells/mm^3^ after ≥2 years of suppressive ART.	8
**Zhao *et al.*,** 2024 [10]	China	Retrospective Cohort	385 (14.29)	41.33 ± 13.39	CD4^+^ T cell count < 200 cells/μl and HIV viral load < 50 copies/ml after 24 months of ART.	9
**Fan *et al.*,** 2023 [1]	China	Retrospective Cohort	150 (29.59)	64.67 ± 4.49	CD4^+^ T cell count below 350 cells/μl at 2 years after ART initiation, with undetectable plasma HIV RNA.	9
**Kacker *et al.*,** 2023 [48]	India	Retrospective Cohort	48 (9.68)	NR	• A return to or fall below the pretherapy (baseline) CD4 at least after 6 months of therapy. • A 50% decline from the on-treatment peak value (if known). • A persistent CD4 count of fewer than 100 cells/mm^3^ after 12 months of therapy.	8
**Taramasso *et al.*,** 2023 [49]	Italy	Retrospective Cohort	5 (1.54)	51 ± 13.4	• CD4 level increase of > 20% from baseline after 36 months of treatment, • CD4 level of > 200 cells/mm^3^ after 36 months of treatment, or CD4 level of > 500 cells/mm^3^ in 36 months.	9
**Wen *et al.*,** 2023 [50]	China	Retrospective Cohort	44 (15.22)	NR	CD4^+^ T-cell count of <200 cells/mL at the 360th (±30) day after HAART initiation.	9
**Zhang *et al.*,** 2023 [51]	China	Retrospective Cohort	549 (20.5)	NR	CD4^+^ T cell counts < 350 cells/μL at 2 years after cART initiation, with an undetectable plasma VL (< 50 copies/mL).	9
**Chen *et al.*,** 2022 [52]	China	Retrospective Cohort	100 (52.36)	38.01 ± 7.7	CD4^+^ T cell count < 350 cells/μl after more than 3 years of treatment.	8
**Fiseha *et al.*,** 2022 [53]	Ethiopia	Retrospective Cohort	160 (36.87)	35 ± 10.47	An increase of >100 cells/mm³ from baseline or achieving an absolute CD4^+^ cell count >500 cells/mm³ at 12 months after ART initiation.	9
**Lei *et al.*,** 2022 [54]	China	Retrospective Cohort	222 (100)	NR	CD4^+^ cell count of less than 200 cells/μl despite receiving highly active antiretroviral therapy (HAART) for over 12 months.	9
**Lembas *et al.*,** 2022 [55]	Poland	Retrospective Cohort	58 (85.29)	NR	Patients who failed to achieve a significant increase in CD4 count, specifically “CD4 < 200 cells/µL,” was a criterion tracked along with ongoing antiretroviral therapy for at least 5 years.	8
**Noiman *et al.*,** 2022 [3]	Multi-countries	Retrospective Cohort	193 (10.82) NHS 254 (25.81) AFRICOS	NHS 36.83 ± 7.17 AFRICOS 40.43 ± 9.62	An absolute CD4 measure of < 350 cells/µl at the time of sustained viral suppression (VS) is defined as achieving and maintaining a viral load < 400 copies/ml for at least two years after starting treatment.	9
**Bayarsaikhan *et al.*,** 2021 [56]	Mongolia	Retrospective Cohort	20 (12.05)	NR	Failure to achieve a CD4 T cell count recovery of more than 50 cells/µL or not reaching a CD4 count of 500 cells/µL after 36 months of antiretroviral therapy.	7
**Han *et al.*,** 2020 [57]	Thailand	Retrospective Cohort	29 (7.73)	38.67 ± 7.8	CD4^+^ T cell count less than 200 cells/mm^3^ at 3 years after initiating ART despite achieving viral suppression with an HIV RNA level of <400 copies/mL.	9
**Li *et al.*,** 2019 [58]	China	Retrospective Cohort	88 (54.66)	NR	• CD4^+^ T cells counts < 200 cells/μL after 2 years HAART is described as Immunological non-responders. • CD4^+^ T cells count 200 ~ 500 cells/μL after 2 years. HAART is described as Immunologically inadequate responders.	9
**Gunda *et al.*,** 2017 [59]	Tanzania	Retrospective Cohort	289 (39.26)	NR	CD4 count of less than 350 cells/μl after 48 months of treatment with HAART.	8
**He *et al.*,** 2016 [60]	China	Retrospective Cohort	769 (83.77)	NR	Inability to recover to specified CD4^+^ counts (≥ 200, 350, or 500 cells/μL) despite being on HAART for at least **1 year** and achieving sustained maximum virological suppression (viral load < 50 copies/mL) by 9 months after starting HAART.	9
**Engsig *et al.*,** 2010 [61]	Denmark	Retrospective Cohort	236 (81.10)	38.33 ± 9.85	CD4 cell count ≤ 200 cells/μL after the three-year suppressed period.	9
**Yeregui *et al.*,** 2022 [16]	Spain	Case-Control	22 (21.8)	NR	CD4^+^ T cell counts below 250 cells/μL after 144 weeks of ART.	8
**Qian *et al.*,** 2021 [62]	China	Case-Control	10 (50)	NR	CD4^+^ T Cell count rises after 2 years of < 100 cells/μl of ART (viral loads < 20 copies/ml).	7
**Yeregui *et al.*,** 2020 [63]	Spain	Case-Control	108 (47.79)	NR	CD4^+^ T cell count < 250 cells/μl after 48 weeks of ART.	8
**Garcia *et al.*,** 2019 [64]	Spain	Case-Control	134 (32.52)	NR	CD4^+^ T cell count below 200 cell/μl after 2 years of treatment using combination ART.	8
**Restrepo *et al.*,** 2019 [15]	Spain	Case-Control	134 (32.52)	NR	CD4^+^ T-cell count < 200 cells/µL after successful viral control for 2 years with cART.	8
**Coelho *et al.*,** 2018 [65]	Brazil	Case-Control	67 (38.07)	NR	CD4^+^ T cell percentage not achieving 30% for two consecutive measurements during follow-up.	8
**Younes *et al.*,** 2018 [66]	Russia-US	Case-Control	16 (34.78)	NR	CD4^+^ T cell counts below 350 cells/μl after at least 2 years of cART with virologic control.	8
**El-Beeli *et al.*,** 2016 [13]	Oman	Case-Control	24 (58.54)	NR	CD4^+^ T-cell count <500 with an increase of less than 50–150 cells/mm³ per year.	8
**Teshome *et al.*,** 2014 [67]	Ethiopia	Case-Control	134 (50.00)	NR	CD4 count declined by 50% from its peak value, persistently below 100 or below the baseline count.	7
**Soria *et al.*,** 2011 [14]	Italy	Case-Control	50 (32.5)	NR	CD4^+^ T-cell counts persistently below 200 cells/mm³ despite achieving and maintaining an undetectable viral load through therapy.	8
**Indrati *et al*.,** 2025 [68]	Bandung, Indonesia	Cross-sectional	15 (37.50)	35 ± 6,5	INR was defined as failure to achieve CD4+ T-cell counts ≥350 cells/mm³ in PLWH on ART with viral suppression (<40 copies/mL).	4
**Rins-Lozano *et al*.,** 2025 [69]	Spain	Cross-sectional	53 (33.33)	47 ± 4,5	INR was defined as a CD4+ T-cell count <500 cells/mm³ after two years of effective ART despite an undetectable viral load.	5
**Tian *et al*.,** 2025 [70]	China	Cross-sectional	32 (45.71)	44,5 ± 6,81	INR was defined as an average of the last two CD4+ T-cell counts <200 cells/µL after 24 months of ART.	7
**Muñoz-Muela *et al*.,** 2024 [71]	Spain	Cross-sectional	53 (33.33)	47 ± 4,5	INR was defined as a CD4+ T-cell count increase <200 cells/μL. A healthy control (HC) group was also included.	6
**Tang *et al*.,** 2024 [72]	China	Cross-sectional	171 (50.00)	44 ± 3,17	INR was defined as persistent CD4+ T-cell counts <200 cells/µL.	4
**Nabatanzi *et al.*,** 2023 [18]	Uganda	Cross-sectional	40 (68.97)	NR	CD4 T cell counts < 500 cells/mL at 12 years of treatment.	8
**Ferrari *et al.*,** 2022 [73]	Multi-countries	Cross-sectional	14 (48.27)	NR	People living with HIV who have a CD4^+^ T cell count < 350 cells/μL after at least 2 years of suppressive antiretroviral therapy (ART).	8
**Lao *et al.*,** 2022 [19]	China	Cross-sectional	57 (42.22)	NR	The last CD4^+^ T counts increased to less than 200 cells/μL after 48 weeks of treatment.	8
**Gelba *et al.*,** 2020 [74]	Ethiopia	Cross-sectional	48 (24.62)	NR	Increase of <50 cells/μL after HAART treatment after 6 months of treatment.	9
**Masson *et al.*,** 2018 [75]	Australia	Cross-sectional	14 (35.89)	NR	CD4^+^ T-cell count of <500 cells/mL despite at least 3 years on cART.	7
**Sun *et al.*,** 2017 [76]	China	Cross-sectional	Not clear	NR	Late ART initiation is defined as a CD4 cell count <200 cells/mm³ or having a clinical AIDS diagnosis prior to ART initiation.	9
**Nguyen *et al.*,** 2016 [77]	US	Cross-sectional	13 (38.2)	NR	CD4^+^ T cell count ≤ 350 cells/µL in their clinical history after 2 years of ART.	8

CD, cluster of differentiation; cART, combination antiretroviral therapy; HAART, highly active antiretroviral therapy; HIV, human immunodeficiency virus; INR, immunological non-response; NOS, Newcastle–Ottawa Scale; NR, not reported; SD, standard deviation; VL, viral load.

### Data source and search strategy

A relevant literature search was conducted using multiple electronic databases such as PubMed, Web of Science, Cochrane Library, ProQuest, and Science Direct for articles up to 31^st^ December 2025. For each database, we used search strategies that focused on the keywords “Human Immunodeficiency Virus Infection” or “HIV Infection” or “acquired immunodeficiency syndrome” or AIDS, and “Immunological nonresponse” or INR. The search strategies were described using Boolean operators and consisted of Medical Subject Heading (MeSH) terms related to the topic [78] (S2 File). The gray literature was also searched to minimize the impact of publication bias, as relying solely on database searches may not provide a sufficiently comprehensive or rigorous overview.

### Study selection

All retrieved studies from the selected databases were imported to Rayyan.ai, an online platform designed to facilitate the study selection process in systematic reviews [79]. All the duplication studies were checked and resolved prior to the study selection. Five reviewers (BER, YTNS, SQK, SMS, and FRN) independently screened all titles and abstracts from the literature search results and evaluated them based on the eligibility criteria. Any emerging discrepancies were resolved by consensus among all authors. The PRISMA flow chart transparently recorded the study selection process [80] (Fig 1).

**Fig 1:**
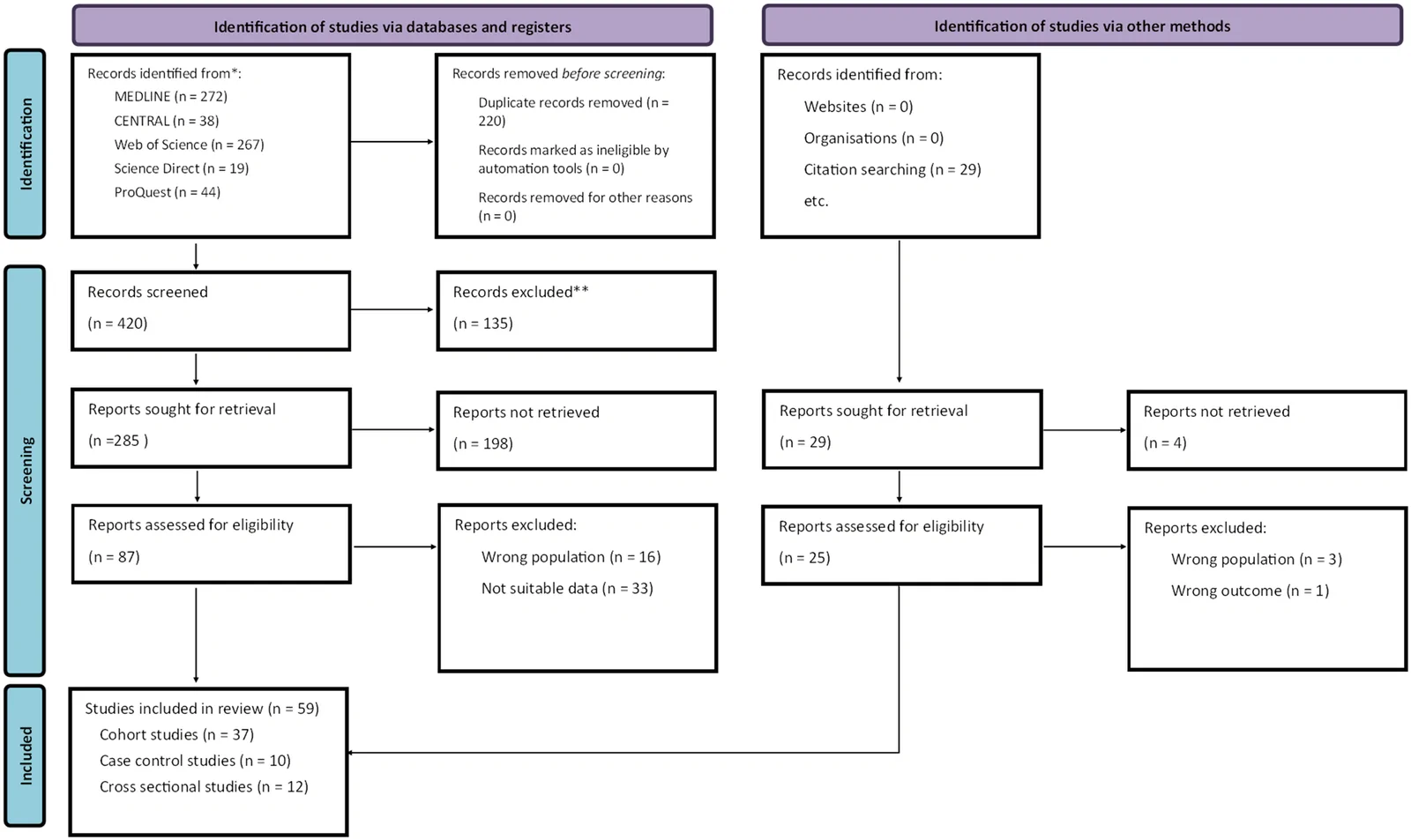
PRISMA flow diagram.

### Data extraction

All the reviewers underwent calibration training in extracting data from the included studies, following the recommendations of the *Cochrane Handbook for Systematic Reviews of Intervention* [28], to ensure consistency and accuracy. The following data for characteristics of included studies were: first author’s name, year of publication, country, study design, sample size, sex, and INR definition. Demographical variables included participants’ mean or median age, sex, route of HIV transmission, and education background. All reported HIV transmission routes were extracted as presented in the primary studies. For quantitative synthesis, each transmission-route category was analyzed separately, and individual studies could therefore contribute data to more than one transmission-route subgroup. Quantitative pooling was performed only for categories with sufficient and comparable data stratified by INR and IR status. We decided to define higher education as a level where the participants had high school and above study experiences, e.g., college or graduate school, while lower education is a level where the participants had below high school experience, e.g., elementary school or middle school. Clinical factors included participants’ co-infections (e.g., hepatitis C, tuberculosis), body mass index, HIV stage, baseline CD4^+^ T cells level and nadir CD4^+^ T cells level. For an additional exploratory analysis of the relationship between sex, parenteral transmission, and viral co-infections, we extracted study-level prevalence of injection drug use (IDU), hepatitis C virus (HCV) co-infection, and hepatitis B virus (HBV) co-infection from studies contributing to the sex meta-analysis. We also sought sex-specific INR data stratified by IDU, HCV, and HBV status to assess whether participant-level stratified analyses were feasible. When cross-stratified data were not explicitly reported, they were considered unavailable and were not imputed. In addition to primary demographic and clinical data, secondary outcome data were extracted to evaluate the biomarker associated with INR. The summarized biomarker findings will be illustrated using Biorender.com. All authors contributed to analyzing the extracted data, and any disagreements or discrepancies were resolved through open discussion among all contributors.

### Quality assessment

Five authors (BER, YTNS, SQK, SMS, FRN) independently assessed the risk of bias for each study, following the Cochrane Handbook for Systematic Reviews of Interventions guidelines. Any disagreements were resolved through discussion among all authors. Additionally, the methodological quality of all included studies was independently evaluated using the Newcastle-Ottawa Scale (NOS), which evaluates three domains: selection, comparability, and exposure, to ensure the robustness of the findings [81]. Study quality was rated as good (scores 7–9), moderate (4–6), or low (0–3), with any differences in assessment settled collaboratively through discussion. The item-level NOS assessments are provided separately for cohort studies (S1 Table in S1 File), case-control studies (S2 Table in S1 File), and cross-sectional studies (S3 Table in S1 File).

### Data synthesis and statistical analysis

All dichotomous and continuous outcome data were synthesized quantitatively. Pooled effect estimates for dichotomous outcomes were reported as odds ratios (ORs), while continuous outcomes were expressed as either mean differences (MD) or standardized mean differences (SMD), each with corresponding 95% confidence intervals (CIs). Continuous data reported as median and range were converted to mean and standard deviation using the method described by Hozo *et al* [82]. Given the anticipated heterogeneity across studies in terms of populations, definitions of INR, and methodologies, a random-effects model (REM) was employed for all meta-analyses using forest plots [83]. The heterogeneity level caused by variability in data was checked by Inconsistency Index (I^2^) and the probability value (p-value), with I² values >50% indicating moderate to high heterogeneity [84]. All statistical tests for this meta-analysis were conducted using R version 4.3.2. To explore whether between-study variation in the association between sex and INR was related to parenteral HIV transmission or viral co-infection burden, we performed post hoc exploratory random-effects meta-regression among studies contributing to the sex meta-analysis for which the relevant study-level data were available. The log-transformed study-specific effect estimate for male versus female sex was used as the dependent variable. Study-level prevalence of IDU, HCV co-infection, and HBV co-infection were evaluated separately as continuous moderators and were scaled per 10-percentage-point increase in prevalence. Between-study variance was estimated using restricted maximum likelihood, with Knapp–Hartung adjustment for statistical inference. The proportion of estimated between-study variance explained by each moderator was summarized using R². Because of the limited number of studies and incomplete overlap in moderator availability, the three moderators were not entered simultaneously into a multivariable meta-regression model. A participant-level stratified analysis according to IDU or viral co-infection status was also considered. Where sufficient data were available, we planned pooled stratified analyses of the association between sex and INR according to IDU status and viral co-infection status (HCV and HBV). Subgroup analysis was performed to evaluate coinfection types and transmission route types as the risk factors of INR. We also conducted a subgroup analysis on other outcomes based on the definition of INR or the CD4^+^ T cell cutoff. However, if the number of included studies for quantitative analysis is small, sensitivity tests and subgroup analysis were not performed.

### Sensitivity analyses and publication bias assessment

The robustness of the findings was tested through sensitivity analyses by repeating the primary meta-analysis and making alternative decisions for ambiguous outcome values [85]. In addition, a sensitivity analysis was conducted by excluding studies identified as outliers based on a visual inspection of the forest plot and funnel plot. Specifically, one study on the age variable was excluded from the sensitivity analysis because it was identified as an outlier. Potential publication bias was assessed using funnel plots generated using R version 4.3.2. An asymmetrical funnel plot shape was interpreted as indicative of potential bias across studies, whereas a symmetrical shape indicates the absence of bias across studies. We also conducted Egger’s test to confirm the presence and significance of publication bias based on the funnel plot results. The interpretation of funnel plots followed guidelines from the *Cochrane Handbook for Systematic Reviews of Interventions* [28].

## Results

### Study identification

A systematic search was conducted across MEDLINE, CENTRAL, Web of Science, ScienceDirect, and ProQuest, yielding 640 records. After removal of 220 duplicate records, 420 records were screened, of which 135 were excluded. A total of 285 reports were sought for retrieval, and 198 reports could not be retrieved, leaving 87 reports for eligibility assessment. Of these, 49 reports were excluded because of an ineligible population (n = 16) or unsuitable data (n = 33), resulting in 38 studies from database and register searches being included in the review. In addition, 29 records were identified through citation searching. Four reports could not be retrieved, leaving 25 reports for eligibility assessment. Four reports were subsequently excluded because of an ineligible population (n = 3) or an ineligible outcome (n = 1), resulting in 21 additional studies. Overall, 59 studies were included in this systematic review, comprising 37 cohort studies, 10 case-control studies, and 12 cross-sectional studies. The PRISMA flow diagram of the study selection process is presented in Fig 1.

### Study characteristics

The included studies were conducted in China, the Netherlands, Italy, India, Brazil, Thailand, Denmark, Switzerland, Indonesia, the United States, Mongolia, Poland, Portugal, Tanzania, Ethiopia, Spain, Russia, Oman, Uganda, and Australia, and were published between 2005 and 2025. Across the included studies, a total of 19,511 participants were classified as having INR. The characteristics of the 59 included studies are summarized in Table 1. All included studies had a Newcastle–Ottawa Scale (NOS) score of ≥5 and were considered to have a moderate-to-low risk of bias. Thirty-seven cohort studies contributed to the quantitative analyses of demographic factors. The corresponding forest and funnel plots are provided in S1–S9 Figs in S1 File. Twenty-six studies reported associations between biomarkers and the INR phenotype

### Data analysis

#### Demographic factors associated with INR.

**Sex:** Thirty-two independent cohorts contributed to the analysis of the association between sex and INR. The pooled analysis showed a statistically significant association between male sex and INR [OR 1.33, 95% CI 1.12–1.58; p = 0.0013], with substantial between-study heterogeneity (I² = 66.7%) (Table 2 and S1 Fig in S1 File). To explore potential sources of heterogeneity in the association between sex and INR, additional study-level meta-regression analyses were performed. Study-level IDU prevalence was available for 10 unique cohorts contributing to the sex analysis. Higher IDU prevalence was significantly associated with a smaller male-versus-female effect estimate (β = −0.113 per 10-percentage-point increase, 95% CI −0.183 to −0.042, p = 0.006). Within these studies, inclusion of IDU prevalence reduced the estimated between-study variance by 93.9%. HCV prevalence was available for 14 studies. A higher HCV prevalence was also associated with a smaller male-versus-female effect estimate; however, this moderator effect did not reach statistical significance (β = −0.108 per 10-percentage-point increase, 95% CI −0.271 to 0.055, p = 0.175), with 16.8% of the estimated between-study variance explained. HBV prevalence was available for 13 studies and was not associated with variation in the sex effect (β = 0.116 per 10-percentage-point increase, 95% CI −0.500 to 0.731, p = 0.687; R² = 0%) (S4 Table in S1 File). Sex-specific INR data stratified by IDU, HCV, or HBV status were insufficiently reported in the primary studies; therefore, participant-level pooled stratified analyses could not be performed.

**Table 2:** Pooled effect estimate demographic and clinical factors influencing immunological non-response.

Factors	Definition	Number of Studies	Odds Ratio or Mean Difference (95% CI)	Analysis Model	Heterogeneity		Overall Effect	
					*p*-value	I^2^ (%)	*Z-*score	*p*-value
Sex	Male vs Female	32	OR 1.33 (1.12–1.58)	REM	<0.0001	66.7	3.21	0.0013
Age	Age on admission (INR group vs IR group)	28	MD 2.61 (1.42–3.81)	REM	< 0.0001	97.6	4.3	< 0.0001
Transmission Route	Heterosexual (INR group vs IR group)	17	OR 0.97 (0.71–1.33)	REM	<0.0001	85.7	−0.2	0.8379
	Homosexual (INR group vs IR group)	17	OR 0.64 (0.52–0.80)	REM	0.0002	63.9	−4.05	< 0.0001
	IV drug use (INR group vs IR group)	13	OR 1.67 (1.15–2.42)	REM	0.002	61.2	2.68	0.0074
Education Background	Lower (Primary high school or lower) vs Higher (High School or higher)	4	OR 1.15 (0.88–1.50)	Fixed-effect model (FEM)	0.0046	77	1.03	0.3038
Coinfection	Hepatitis B (INR group vs IR group)	14	OR 1.30 (1.08–1.57)	FEM	0.1935	24.1	2.74	0.0061
	Hepatitis C (INR group vs IR group)	15	OR 1.85 (1.50–2.28)	FEM	0.2535	17.9	5.77	< 0.0001
	Tuberculosis (INR group vs IR group)	7	OR 1.77 (1.41–2.21)	FEM	0.011	63.8	4.96	< 0.0001
Body Mass Index	Underweight vs. Normal	5	OR 1.30 (0.81–2.10)	REM	< 0.0001	88.9	1.09	0.2766
HIV Stage	Stage III-IV vs I-II	5	OR 2.66 (2.34–3.02)	FEM	0.0004	80.6	15.05	< 0.0001
Baseline CD4^+^ T Cells Count	INR group vs IR group	15	SMD −3.82 (−4.94,-2.70)	REM	0	99.3	−6.7	< 0.0001
Nadir CD4^+^ T Cells Count	INR group vs IR group	10	SMD −1.69 (−2.69,-0.68)	REM	< 0.0001	99.1	−3.29	0.001

For transmission-route analyses, individual studies could contribute to more than one category; therefore, subgroup study counts should not be summed to determine the number of unique studies. Seventeen unique studies contributed to the quantitative analysis. Blood-transfusion-acquired HIV was reported in two studies, but only one provided INR-versus-IR stratified data, precluding quantitative pooling. Mother-to-child transmission was not available as a separately extractable comparative category. INR, immunological non-response; IR, immunological response; CI, confidence interval; OR, odds ratio; MD, mean difference; SMD, standardized mean difference; REM, random-effects model; FEM, fixed-effect model; I², I-squared statistic; CD, cluster of differentiation.

**Age:** Twenty-eight studies reported mean age and standard deviation separately for the INR and IR groups. Age was analyzed as a continuous variable. The results are shown in Table 2 and S2 Fig in S1 File. Participants with INR were significantly older than those with IR [MD 2.61 years, 95% CI 1.42–3.81; p < 0.0001; I² = 97.6%].

**Transmission route:** Seventeen unique studies provided extractable comparative data on HIV transmission routes. Sufficient INR-versus-IR data were available for heterosexual and homosexual transmission in 17 studies and for intravenous drug use in 13 studies. Blood-transfusion-acquired HIV was reported in two studies; however, only one provided data stratified by INR and IR status, precluding quantitative pooling. Mother-to-child transmission was not available as a separately extractable comparative category. Therefore, the quantitative meta-analysis was restricted to heterosexual transmission, homosexual transmission, and intravenous drug use (Table 2 and S3 Fig in S1 File). Homosexual transmission was statistically significantly associated with lower odds of INR [OR 0.64, 95% CI 0.52–0.80; p < 0.0001; I² = 63.9%], whereas intravenous drug use was statistically significantly associated with higher odds of INR [OR 1.67, 95% CI 1.15–2.42; p = 0.0074; I² = 61.2%]. No statistically significant association was observed for heterosexual transmission [OR 0.97, 95% CI 0.71–1.33; p = 0.8379; I² = 85.7%].

**Education:** Four studies [12,31,44,53] reported participants’ educational attainment at baseline. The results are shown in Table 2 and S4 Fig in S1 File. The pooled analysis showed no significant association between lower educational attainment and INR [OR 1.15, 95% CI 0.88–1.50; p = 0.3038; I² = 77.0%].

#### Clinical factors associated with INR.

**Coinfection:** Thirty-six studies reported data on co-infections among PLWH. Co-infections were categorized as hepatitis A, hepatitis B, hepatitis C, and tuberculosis. The results are shown in Table 2 and S5 Fig in S1 File. Overall, co-infection was statistically significantly associated with higher odds of INR [OR 1.58, 95% CI 1.41–1.77; p < 0.0001; I² = 37.5%]. In subgroup analyses, significant associations with INR were observed for hepatitis B co-infection [OR 1.30, 95% CI 1.08–1.57; p = 0.0061; I² = 24.1%], hepatitis C co-infection [OR 1.85, 95% CI 1.50–2.28; p < 0.0001; I² = 17.9%], and tuberculosis [OR 1.77, 95% CI 1.41–2.21; p < 0.0001; I² = 63.8%]. Only one study reported hepatitis A co-infection; therefore, a pooled effect estimate was not calculated.

**Body Mass Index:** Five studies evaluated the association between body mass index (BMI) and INR. The quantitative analysis compared participants who were underweight with those of normal weight. Other BMI-category comparisons were not pooled because insufficient comparable data were available. The results are shown in Table 2 and S6 Fig in S1 File. Underweight status was not significantly associated with INR [OR 1.30, 95% CI 0.81–2.10; p = 0.2766; I² = 88.9%].

**HIV stage:** The included studies used different HIV disease-staging systems, including the Centers for Disease Control and Prevention (CDC) [42,49,57], Fiebig [35], and World Health Organization (WHO) classifications [52,53,59]. Because WHO stage data were sufficiently comparable for quantitative pooling, three cohort studies reporting WHO stage were included in the meta-analysis [52,53,59]. The results are shown in Table 2 and S7 Fig in S1 File. Advanced HIV stage (III–IV vs I–II) was statistically significantly associated with higher odds of INR [OR 2.66, 95% CI 2.34–3.02; p < 0.0001; I² = 80.6%].

**Baseline CD4**^**+**^ **T cell count:** Fifteen studies reported baseline CD4⁺ T-cell counts, which were analyzed as continuous variables. The results are shown in Table 2 and S8 Fig in S1 File. Baseline CD4 ⁺ T-cell counts were significantly lower in the INR group than in the IR group [SMD −3.82, 95% CI −4.94 to −2.70; p < 0.0001; I² = 99.3%].

**Nadir CD4**^**+**^ **T cells count:** Ten studies reported nadir CD4⁺ T-cell counts, which were analyzed as continuous variables. The results are shown in Table 2 and S9 Fig in S1 File. Nadir CD4⁺ T-cell counts were significantly lower in the INR group than in the IR group [SMD −1.69, 95% CI −2.69 to −0.68; p = 0.001; I² = 99.1%].

**Markers associated with INR:** We identified evidence on 25 genetic markers, 17 protein or metabolic markers, and 10 other markers associated with the INR phenotype. These markers and their reported associations are summarized in Table 3.

**Table 3:** Predictive markers for immunological non-response.

Genetic Marker	References	Marker Role in INR	Sample Size	Key Finding	Genotyping Method
HLA B13	Pereira *et al.*, 2022 [12]	Association with immunological non-response after ART.	4	HLA-B13 showed higher estimated odds of INR, but the association was not statistically significant [OR 4.06, 95% CI 0.45–36.62].	Polymerase chain reaction sequence-specific oligonucleotide (PCR-SSO)
HLA B35	Pereira *et al.*, 2022 [12]	Association with immunological non-response after ART.	42	HLA-B35 was statistically significantly associated with higher odds of INR [OR 1.86, 95% CI 1.08–3.18].	PCR-SSO
HLA B39	Pereira *et al.*, 2022 [12]	Association with immunological non-response after ART.	19	HLA-B39 was statistically significantly associated with higher odds of INR [OR 3.33, 95% CI 1.33–8.69].	PCR-SSO
*HLA A68*	El-Beeli *et al.*, 2017 [13]	*HLA A68* is linked to weaker or less durable CD8+ T cell responses against HIV.	6	The frequency of HLA-A68 was significantly higher in the INR group than in the IR group (p = 0.025). Participants carrying the HLA-A68 allele were 2.37 times more likely to exhibit poor immune recovery (95% CI, 1.37–4.13).	Polymerase chain reaction with sequence-specific primers (PCR-SSP)
*HLA B15*	El-Beeli *et al.*, 2017 [13]	*HLA B15* is linked to the lack of response to antiretroviral therapy among patients who have already had AIDS condition.	5	A significant difference was observed in the frequency of HLA-B15, which was present in 20.8% of the INR group compared with 2.4% of the IR group (p = 0.023). The odds of inadequate response to cART are 2.5 times (RR = 2.59; 95% CI = 1.55, 4.33) higher among AIDS patients who carry HLA-B15. HLA-B15 is responsible for nearly two-thirds of poor responses to cART (EFe% = 61.36%; 95% CI = 35.33, 76.91.	PCR-SSP
*KIR2DL3*	Soria *et al.*, 2011 [14]	A polymorphic set of molecules that modulate natural killer (NK) cell activity. Its reduced expression or absence in individuals with INR may lead to impaired NK-cell function, increased immune activation, and hindered immune recovery during ART.	50	The KIR2DL3 allele was independently associated with a reduced risk of INR, including after adjustment for nadir CD4+ T-cell count [OR (95% CI), 0.30 (0.12–0.74); p = 0.009].	PCR-SSP
KIR2DS2	Soria *et al.*, 2011 [14]	Activating killer cell immunoglobulin-like receptor (KIR) involved in the regulation of NK-cell activity.	50	KIR2DS2 was more frequent in the INR group than in full responders (74.0% vs 54.8%; crude OR = 2.35, 95% CI: 1.06–5.27; p = 0.03); however, the association was no longer statistically significant after adjustment for nadir CD4⁺ T-cell count (adjusted OR = 1.93, 95% CI: 0.84–4.46; p = 0.12).	PCR-SSP
Compound genotype *HLA-C1*+/KIR2DL3+	Soria *et al.*, 2011 [14]	Specific epistatic interactions between HLA- and KIR-encoding genes were found to be associated with a delay in disease progression and a stronger viral control in HIV-infected cART-naive individuals.	50	The functional compound genotype HLA-C1+/KIR2DL3+, even at multivariable analysis, when adjusted for nadir CD4^+^ T-cell count, was associated with reduced risk of INR status [OR 0.34 (0.13–0.88), *p* = 0.03].	PCR-SSP
HLA-C1⁺/KIR2DS2^−^/KIR2DL3⁺	Soria *et al.*, 2011 [14]	KIR–HLA compound genotype associated with a reduced risk of immunological non-response.	50	The HLA-C1⁺/KIR2DS2^−^/KIR2DL3⁺ combination was less frequent in the INR group than in full responders (10.0% vs 34.6%). It was associated with a reduced risk of INR in both crude analysis (OR = 0.15, 95% CI: 0.04–0.57; p = 0.003) and analysis adjusted for nadir CD4 ⁺ T-cell count (adjusted OR = 0.16, 95% CI: 0.04–0.64; p = 0.01).	PCR-SSP
*CCR5 (D32) allele*	Carvalho-Silva *et al.*, 2020 [17]	Expresses a receptor that is involved in the co-activation of CD4^+^ T-lymphocytes, and, thus, it could be involved in the immunological reconstitution.	12	Heterozygosity for CC chemokine receptor (CCR5Δ32) polymorphism was associated with immunological reconstitution failure in the logistic regression analysis, being linked with a five-times higher risk of poor immune recovery (OR = 5.873, 95%CI = 1.204–28.633, *p* = 0.028).	Conventional PCR followed by agarose gel electrophoresis
rs2430561 *IFNγ*	Garcia *et al.*, 2019 [64]	The A allele variant reduces interferon (IFNγ) expression.	134	rs2430561 IFNγ was significantly associated with INR [OR 1.83 (1.10–3.05)].	Matrix-assisted laser desorption/ionization time-of-flight mass spectrometry (MALDI-TOF MS)
rs1493013 *IL15*	Garcia *et al.*, 2019 [64]	T-cell homeostasis.	134	rs1493013 IL15 presented a clear trend for association with INR status [OR 1.55 (0.99–2.43)].	MALDI-TOF MS
rs2243191 *IL19*	Garcia *et al.*, 2019 [64]	The allele C variant was associated with post-transcriptional regulation of *IL19* through RNA-binding protein ELAVL1.	134	rs2243191 IL19 was significantly associated with INR status [OR 2.22 (1.07–4.59)].	MALDI-TOF MS
rs1799764 *CCR2 AG genotype*	Restrepo *et al.*, 2019 [15]	The rs1799864 variation results in a valine to isoleucine substitution at position 64 in the CCR2 protein, which may alter receptor function and influence immune cell trafficking, potentially impacting CD4^+^ T-cell recovery.	134	rs1779864 *CCR2 AG genotype* showed a trend to be associated with INR status [OR 1.50 (0.91–2.58), *p* = 0.10].	MALDI-TOF MS
rs1801157 *CXCL12*	Restrepo *et al.*, 2019 [15]	Expresses CXC chemokine ligand (CXCL12, which activates and/or induces migration of hematopoietic progenitor and stem cells, endothelial cells, and most leukocytes.	134	rs1801157 *CXCL12 TT genotype* showed a trend to be associated with INR status [OR 2.40 (0.96–6.13), *p* 0.05].	MALDI-TOF MS
	Yeregui *et al.*, 2020 [63]		98	Regarding rs1801157 CXCL12, the results suggested a potential association between CXCL12 expression and the immune response to cART (*p* = 0.065).	Kompetitive Allele-Specific PCR (KASP-PCR)
rs2280788 *CCL5*	Yeregui *et al.*, 2020 [63]	Expresses C-C motif chemokine ligand 5 (CCL5), which directs T-cell migration to damaged or infected regions and controls T-cell differentiation through type 1 of T helper (Th1) cell recruitment.	103	rs2280788 CCL5 was strongly related to plasma MIP-b in the INR population (*p* = 0.041).	KASP-PCR
rs1799864_814 *CCR2*	Yeregui *et al.*, 2020 [63]	Expresses CCR2, which regulates the mobilization of monocytes from bone marrow to the inflammatory sites.	100	*rs1799864_814 CCR2* was significantly related to the plasma RANTES concentration in the INR population, corroborating the binding of this chemokine to the CCR2 receptor (*p* < 0.05).	KASP-PCR
rs333_814 (D32) *CCL5*	Yeregui *et al.*, 2020 [63]	Expresses CCL5, which directs T-cell migration to damaged or infected regions and controls T-cell differentiation through type 1 of T helper (Th1) cell recruitment.	100	rs333_814 (D32) *CCR5* determines the baseline plasma MIP-a concentration in the INR population (*p* < 0.05).	KASP-PCR
rs2069762 *IL2 C/C genotype*	Coelho *et al.*, 2018 [65]	Regulates T and B lymphocyte proliferation.	67	*IL2* rs2069762 C/C genotype had an eight-fold higher probability of achieving immunologic response when compared to the A/A genotype [HR 8.60 (2.06–35.89), *p* = 0.003].	Multiplexed allele-specific oligo ligation & PCR
rs2243250 *IL4*	Coelho *et al.*, 2018 [65]	Immunoregulation	67	not significantly associated with the probability of immune response on multivariate analysis.	Multiplexed allele-specific oligo ligation & PCR
rs1128503 *ABCB1 A/A genotype*	Coelho *et al.*, 2018 [65]	Membrane transport, antiretroviral drugs efflux.	67	ATP-binding cassette subfamily B1 *(ABCB1)* rs1128503 A/A genotype was associated with a 71% lower chance of achieving immunologic response when compared with the G/G genotype [HR 0.29 (0.15–0.58), *p* = 0.0004].	Multiplexed allele-specific oligo ligation & PCR
rs4646437 *CYP3A4*	Coelho *et al.*, 2018 [65]	Antiretroviral drugs metabolism.	67	not significantly associated with the probability of immune response on multivariate analysis.	Multiplexed allele-specific oligo ligation & PCR
rs707265 *CYP2B6*	Coelho *et al.*, 2018 [65]	Antiretroviral drugs metabolism.	67	not significantly associated with the probability of immune response on multivariate analysis.	Multiplexed allele-specific oligo ligation & PCR
rs1385129 *SLC2A1*	Masson *et al.*, 2018 [75]	Elevated glucose transporter 1 (Glut1) expression on CD4^+^ T cells, associated with the rs1385129 GG genotype, may lead to increased glucose uptake and metabolic activity, contributing to immune activation and impaired CD4^+^ T-cell recovery.	39	The GG genotype of solute carrier family 2 member 1 (SLC2A1) rs1385129 was significantly associated with higher CD4^+^ Glut1+ T cell percentages in HIV+/cART individuals (*p* = 0.038). Those with the GG genotype had a 4.67-fold increased risk of poor response to therapy compared to GA/AA carriers (*p* = 0.04).	Multiple SNPs Genotyping
rs1130214 *AKT*	Masson *et al.*, 2018 [75]	Elevated Glut1 expression on CD4^+^ T cells, associated with the GT genotype of rs1130214, may lead to increased glucose uptake and metabolic activity, contributing to immune activation and impaired CD4^+^ T-cell recovery.	39	The GT genotype of protein kinase B (AKT) rs1130214 was associated with persistently high CD4^+^ T cell glycolytic activity in HIV-positive cART-treated individuals (*p* = 0.0068). However, rs1130214 did not reach a significant relationship with clinical outcomes in terms of disease progression or treatment response.	Multiple SNPs Genotyping
HIV-1 integrase mRNA (p31)	Tang *et al.,* 2024 [72]	Elevated levels of HIV-1 integrase mRNA (p31) in peripheral blood mononuclear cells (PBMCs) of individuals with INR signify higher transcriptional activity of the HIV-1 virus within these individuals, even under ART, suggesting the virus is more actively replicating or maintaining its presence in individuals with INR.		The INR group exhibited significantly higher levels of HIV-1 integrase mRNA (p31) in peripheral blood mononuclear cells (PBMCs) than the IR group (p = 0.003).	Reverse transcription quantitative polymerase chain reaction (RT-qPCR)
Indole-3-acetyl-beta-1-D-glucoside	Tian, *et al.,* 2025 [70]	Immunomodulatory effects, specifically by inhibiting T-cell activation and suppressing pro-inflammatory cytokines.	32	Indole-3-acetyl-beta-1-D-glucoside (area under the curve value = 0.8931), had the best discriminatory ability. Further experiments showed that Indole-3-acetyl-beta-1-D-glucoside significantly decreased the proportions of CD4+CD57+, effector CD4+, CD4+PD1+, CD8+CD57+, effector CD8+, and CD8+HLA-DR+ T cells. Moreover, mRNA expression analysis showed that Indole-3-acetyl-beta-1-D-glucoside treatment led to a suppression of pro inflammatory cytokines.	16S rRNA gene sequencing and liquid chromatography-mass spectrometry (LC-MS) untargeted metabolomics
Soluble cluster of differentiation 14 (sCD14)	Muñoz-Muela *et al.,* 2024 [71]	Monocyte activation.	35	The plasma concentration of sCD14 was higher in naïve [3225.0 ng/ml (2342.0–4090.0)] than in the pooled HC, IR, and INR groups [2196.0 (1648.0–2759.0), p < 0.001], among which there were no significant differences.	droplet digital polymerase chain reaction (ddPCR)
sCD163	Muñoz-Muela *et al.,* 2024 [71]	sCD163 is linked to viral activity monocytes and monocyte activation and also coupling efficiency inversely correlated to sCD163 reinforcing connection between monocyte activation and mitochondrial dysfunction.	35	The concentrations of sCD163 were the highest in naïve [172.7 ng/ml (117.6–293.0)] followed by INR [111.4 (95.2–136.1), p = 0.005], IR [85.0 (57.2–131.4), p = 0.010 for INR vs. IR] and HC [75.6 (59.4–109.3), p = 0.662 for IR vs. HC].	ddPCR
β2-m	Muñoz-Muela *et al.,* 2024 [71]	Chronic immune activation and inflammation.	35	Higher levels of β2-m (p < 0.001) was found in plasma from naïve compared to treatment-experienced participants. The INR group had higher plasma concentrations of β2-m (p < 0.001) than HC.	ddPCR
Interferon gamma-induced protein 10 (IP-10)	Muñoz-Muela *et al.,* 2024 [71]	IP-10 levels were correlated with monocyte activation, particularly with the expression of GLUT1^high^ showing potential link IP-10, monocyte metabolism, and chronic immune activation.	35	The INR group had higher plasma concentrations of IP-10 (p = 0.014) than HC and higher plasma levels of IP-10 compared to IR (p = 0.065).	ddPCR
					
**Protein or Metabolomic Marker**	**References**	**Marker Role in INR**	**Sample Size**	**Significancy**	**Pathway/Biological Process**
Caspase-1	Lao *et al.*, 2022 [19]	Pyroptosis mediator: Caspase-1 specifically cleaved the linker between the amino-terminal gasdermin-N and carboxy-terminal gasdermin-C domains in gasdermin D (GSDMD), resulting in the gasdermin-N domain, triggering pyroptosis.	57	Caspase-1 cytokine significantly showed a higher level in the INR population [OR 1.846 (0.291–11.691), *p* = 0.046]	Inflammation (Pyroptosis)
	Nabatanzi *et al.*, 2023 [18]		40	Higher percentages of caspase-1 expression from CD4 T cells in peripheral blood of individuals with higher immune activation levels (median, 19.30 [IQR, 7.285–21.5]) relative to individuals with lower immune activation (median, 9.6 [IQR, 6.74–12.10]) (*p* = 0.04).	
IL-1β	Lao *et al.*, 2022 [19]	Recruit and activate other immune cells and induce the synthesis of IL-6, chemokines, and adhesion molecules, amplifying the local and systemic inflammatory response during pyroptosis.	57	IL-1β in this study showed a slightly difference level between the INR and the IR group [OR 1.151 (0.846–1.565), *p* = 0.370]	Inflammation (Pyroptosis)
	Nabatanzi *et al.*, 2023 [18]		40	Higher plasma levels of inflammatory byproduct IL-1β in individuals with higher immune activation levels (median, 8.945 [IQR, 6.398–20.69]) relative to individuals with lower immune activation (median, 5.077 [IQR, 4.416–6.492]; *p* = 0.009).	
IL-18	Lao *et al.*, 2022 [19]	Recruit and activate other immune cells and induce the synthesis of IL-6, chemokines, and adhesion molecules, amplifying the local and systemic inflammatory response during pyroptosis.	57	IL-18 cytokine significantly showed a higher level in the INR population [OR 3.883 (1.692–8.907), *p* = 0001]	Inflammation (Pyroptosis)
	Nabatanzi *et al.*, 2023 [18]		40	Plasma level of IL-18 was comparable among individuals with higher immune activation [median 191.7 (IQR 87.14–219.4)] relative to individuals with lower immune activation [median 131.4 (IQR 104.6–219.4)] *p* = 0.065.	
IL-18Bpa	Nabatanzi *et al.*, 2023 [18]	Binding protein which antagonized IL18.	40	Plasma levels of IL-18BPa were comparable among individuals with higher immune activation relative [median 771.9 (IQR 561–1129)] to individuals with lower immune activation [Median 887.7 (IQR 687.6–1114)] *p* = 0.643.	Inflammation (Pyroptosis)
GSDMD	Lao *et al.*, 2022 [19]	Controls the release of the proinflammatory cytokines and pyroptotic cell death.	57	There were no significant differences observed in the expression of GSDMD between the IR and INR populations [OR 1.541 (0.578–4.112), *p* = 0.3888].	Inflammation (Pyroptosis)
Acylcarnitines	Qian *et al.*, 2021 [62]	Mitochondrial dysfunction through apoptosis and membrane disruption.	10	Acylcarnitine levels were negatively correlated with CD4+ T-cell count in individuals with INR. Specifically, myristoylcarnitine (MC; p = 0.034, r = −0.476), palmitoylcarnitine (PC; p = 0.047, r = −0.450), stearoylcarnitine (SC; p = 0.008, r = −0.574), and oleoylcarnitine (OC; p = 0.007, r = −0.581) showed significant inverse correlations with CD4+ T-cell count.	Mitochondrial Dysregulation
Stromal cell-derived factor 1 (SDF-1)	Yeregui *et al.*, 2020 [63]	Regulates immune system survival through receptors CXCR4 and atypical chemokine receptor (ACKR3).	118	The level of SDF-1 was significantly different between the INR and IR groups at both baseline (*p* = 0.036) and after 48 w of cART (*p* = 0.036), which indicates SDF-1 is not only a prognostic marker of the immune response and disease progression.	Immune Survival Impairment
Monocyte chemoattractant protein-1 (MCP-1)	Yeregui *et al.*, 2020 [63]	Chemoattractant for CD4^+^ T cells.	118	The level of MCP-1 was significantly different between the INR and IR groups (p = 0.001).	Immune Survival Impairment
Peroxisome proliferator-activated receptor-γ coactivator 1α (PGC1α)	Younes *et al.*, 2018 [66]	Regulates mitochondrial biogenesis.	16	Based on the flow cytometry results, cycling Tregs from individuals with INR showed significantly diminished expression of PGC1α, consistent with the decreased mitochondrial mass and oxidative phosphorylation (OXPHOS) in these cells.	Mitochondrial Dysregulation
Mitochondrial transcription factor A (TFAM)	Younes *et al.*, 2018 [66]	Regulates the expression of 13 mitochondrial genes implicated in oxidative phosphorylation.	16	Based on the flow cytometry results, cycling Tregs from individuals with INR showed significantly diminished expression of TFAM, consistent with the decreased mitochondrial mass and OXPHOS in these cells.	Mitochondrial Dysregulation
IL-7	Nguyen *et al.*, 2016 [77]	IL-7-induced STAT5 phosphorylation (P-STAT5) results in the rapid induction of an anti-apoptotic protein, bcl-2, and up-regulation of IL-2Rα (CD25) thus improving the T cell recovery.	11	CD25 induction in response to IL-7 was directly correlated with CD127 expression in the HIV-infected subjects. When assessed independently in the INR and IR groups, the relationships remained positive but did not reach statistical significance (p > 0.05). In contrast, proliferation responses to IL-7 stimulation were poorly correlated with CD127 expression.	Immune Survival Impairment
Adiponectin	Yeregui *et al.*, 2022 [16]	Adiponectin decreases inflammation in macrophages, endothelial tissue, muscle cells, and epithelial cells.	101	Adiponectin was significantly higher in both IR and INR groups compared to controls, suggesting that it represents a marker of low nadir CD4^+^ T cell counts (*p* < 0.05).	Immune Metabolism Impairment
Apelin	Yeregui *et al.*, 2022 [16]	It inhibits the entrance of HIV into human cells by binding to apelin receptor (APLNR). In this sense, both apelin and APLNR were negatively associated with the VL but positively correlated with the CD4^+^ T-cell count, which supports the relation of this adipokine and its receptor to HIV replication.	101	Circulating apelin concentrations were significantly lower in individuals with INR than in controls (*p* = 0.011).	Immune Metabolism Impairment
APLNR	Yeregui *et al.*, 2022 [16]	Immune system regulator and contributes to T cell survival.	101	APLNR was significantly lower in the INR groups, suggesting that it is a predictive marker of poor immune recovery independently related to a low nadir CD4^+^ T cell count (*p* = 0.007).	Immune Metabolism Impairment
zinc-alpha-2-glycoprotein (ZAG)	Yeregui *et al.*, 2022 [16]	ZAG is an immunoregulator glycoprotein associated with better CD4^+^ T-cell reconstitution and may promote anti-inflammatory effects.	101	ZAG was significantly lower in the INR groups, suggesting that it is a predictive marker of poor immune recovery independently related to a low nadir CD4^+^ T cell count (*p* = 0.040).	Immune Metabolism Impairment
Retinol-binding protein 4 (RBP4)	Yeregui *et al.*, 2022 [16]	Vitamin A transporter, which influences T-cell differentiation, antibody production, and mucosal immunity.	101	RBP4 was significantly lower in the INR groups, suggesting that it is a predictive marker of poor immune recovery independently related to a low nadir CD4^+^ T cell count (*p* < 0.001).	Immune Metabolism Impairment
IL-6	Muñoz-Muela *et al.,* 2024 [71]	Healthy controls (HC), the IR group, and the INR group generally showed similar plasma concentrations of IL-6.	35	IL-6 levels in the INR group were approximately twice as high as those in the naïve and IR groups, although statistical significance was reached only when compared with HC (p = 0.048). IL-6 concentrations were weakly correlated with the percentage of non-classical monocytes (ρ = 0.230, p = 0.033).	Inflammation
Parathyroid hormone (PTH)	Rins-Lozano *et al.,* 2025 [69]	Higher levels of PTH secondary to lower vitamin D levels might influence bone material strength index (BMSi).	45	The INR group exhibited higher PTH levels with a median of 50 pg/mL (interquartile range [IQR] 37–63) compared with the IR group (41 pg/mL [IQR 32–54]); P = .034. Moreover, the INR group had significantly lower levels of 25-OH-vitamin D3, with a median of 18.8 ng/mL (IQR 12–33), compared with 27 ng/mL (IQR 17–38) in the IR group (p = 0.045).	Inflammation
High-sensitivity C-reactive protein (hs-CRP)	Rins-Lozano *et al.,* 2025 [69]	hs-CRP is an inflammatory marker linked to poorer bone material through elevated inflammation and immune activation, particularly in individuals with INR, can alter B-cell and T-cell compartments, disrupting the balance of osteoblastic and osteoclastic cytokines. This imbalance can promote osteoclast formation and bone resorption, leading to bone mineral content loss and impaired bone quality.	53	Inflammation, estimated by hs-CRP, was significantly higher in the INR group, with a median level of 0.39 mg/dL (IQR 0.08–0.55), compared with 0.15 mg/dL (IQR 0.04–0.355) in the IR group (p = 0.017). An inverse correlation between hs-CRP levels and BMSi was observed in both groups and was more pronounced in the INR group (Spearman rho −0.406, p = 0.003) than in the IR group (Spearman rho −0.218, p = 0.027).	Inflammation
					
**Other Marker**	**References**	**Marker Role in INR**	**Sample Size**	**Significancy**	**Specific Pathway Involved**
RTE	Guedes *et al.*, 2023 [32]	Immature naïve T cells originating in the thymus, crucial for immune reconstitution.	13	Recent thymic emigrant (RTE) percentage was lower in the INR group (19.5 + − 6.3) compared to the IR group (29.9 + − 11.5), with a statistically significant difference (*p* = 0.038), demonstrating the influence of these cells on immune reconstitution.	T-cell Production Impairment
T-cell receptor excision circle (TREC)	Rb-Silva *et al.*, 2019 [39]	The INR group exhibited significantly lower sj-TREC counts than the IR group at multiple time points during ART, indicating a reduced contribution of thymic activity to CD4+ T-cell recovery.	33	sj-TREC levels were significantly lower in the INR group than in the IR group at 24 months of ART (p < 0.01).	T-cell Production Impairment
Lipopolysaccharide (LPS)	Tian *et al.*, 2023 [46]	LPS in the bloodstream results from microbial translocation due to gut dysbiosis. It contributes to chronic immune dysregulation by overstimulating Toll-like receptor 4 (TLR4), leading to excessive activation of the NF-κB signaling pathway and impairing immune recovery.	28	LPS was significantly higher in the INR group than in the IR group (p = 0.0185). The diagnostic accuracy based on the area under the curve (AUC) values of LPS indicates moderate discrimination ability (AUC = 0.682).	Microbial Translocation
	Merlini *et al.*, 2021 [33]		278	LPS showed no significant association with the clinical progression of people living with HIV (*p* = 0.524).	
Lipopolysaccharide-binding protein (LBP)	Tian *et al.*, 2023 [46]	LBP binds LPS in the bloodstream, facilitating its interaction with CD14 and Toll-like receptor 4 (TLR4), which drives immune dysregulation and chronic inflammation.	28	LBP was significantly associated with the INR group compared with the IR group (p < 0.0001). The diagnostic accuracy based on the AUC values of LBP indicates high discrimination ability (AUC = 0.876).	Microbial Translocation
sCD14	Tian *et al.*, 2023 [46]	sCD14 is a protein released by monocytes and macrophages in response to LPS binding, serving as a co-receptor for TLR4. Elevated sCD14 levels indicate persistent immune activation and can further drive immune exhaustion.	28	sCD14 was significantly associated with the INR group compared with the IR group (p < 0.0001). The diagnostic accuracy based on the AUC values of sCD14 indicates a strong biomarker for distinguishing INR (AUC = 0.883).	Microbial Translocation
	Merlini *et al.*, 2021 [33]		477	sCD14 showed no significant association with the clinical progression of people living with HIV (*p* = 0.821).	
Endogenous endotoxin-core antibody (EndoCAb)	Tian *et al.*, 2023 [46]	EndoCAb are natural antibodies that neutralize circulating LPS, helping to regulate immune responses to microbial translocation. In individuals with INR, persistent microbial translocation results in the depletion of EndoCAb, limiting the body’s ability to control endotoxemia.	28	EndoCAb was significantly increased in the INR group compared with the IR group (p < 0.0001). The diagnostic accuracy based on the AUC values of EndoCAb indicates a good predictive value of INR (AUC = 0.800).	Microbial Translocation
	Merlini *et al.*, 2021 [33]		475	EndoCAb was initially significantly associated with non-infectious events (HR = 0.52, *p* = 0.026). However, the association disappeared after adjusting for confounders (*p* > 0.05).	
Intestinal fatty acid-binding protein (I-FABP)	Tian *et al.*, 2023 [46]	I-FABP is a biomarker of intestinal epithelial integrity. In individuals with INR, persistent gut damage and microbial translocation drive chronic immune activation, which leads to immune exhaustion.	28	No significant difference in I-FABP levels was observed between the INR and IR groups (p > 0.05). The diagnostic accuracy also needs further research to associate it with INR phenotype (AUC = 0.601).	Microbial Translocation
p-cresol sulfate (PCS)	Ferrari et al, 2022 [73]	PCS is produced from tyrosine and phenylalanine by gut bacteria, which is highly correlated with the level of pro-inflammatory cytokine.	14	PCS was significantly 2-fold higher in the INR group (*p* = 0.0017).	Microbial Translocation
IS	Ferrari *et al.*, 2022 [73]	IS is produced from tryptophan by gut bacterial tryptophanase, which can alter the mitochondrial function of PLWH.	14	IS was significantly 1.3-fold higher in the INR group.	Microbial Translocation
NK-cell immunophenotype	Vos *et al.*, 2024 [47]	Alterations in NK-cell abundance and activation phenotype.	62	Absolute NK-cell and NK T-like-cell numbers were lower in INR group, whereas overall NK-cell proportions were comparable. HLA-DR⁺ NK-cell and NK T-like-cell subpopulations were increased in INR with similar trends in the validation cohort, while CD38⁺ NK-cell subsets showed no consistent pattern across cohorts.	NK-cell abundance and activation
CD4/CD8 ratio and CD8⁺ T-cell phenotype	Vos *et al.*, 2024 [47]	Altered CD4/CD8 T-cell homeostasis and CD8⁺ T-cell immunophenotype.	62	Lower pre-cART and most recently measured CD4/CD8 ratios were associated with INR in the discovery cohort (both p < 0.001), but were not significantly validated in the validation cohort. Absolute CD8⁺ naïve, CD8 TC17, and CD8 TC1/17 subsets were reduced in INR group, while CD8⁺ T-cell activation-marker patterns were not consistently reproduced across cohorts.	T-cell homeostasis, immune activation, and immunosenescence

ABCB1, ATP-binding cassette subfamily B1; ACKR, atypical chemokine receptor; AKT, protein kinase B; APLNR, apelin receptor; ART, antiretroviral therapy; AUC, area under the curve; CCR, CC chemokine receptor; CCR2, CC chemokine receptor 2; CCL5, C-C motif chemokine ligand 5; CD4, cluster of differentiation 4; CD8, cluster of differentiation 8; CI, confidence interval; cART, combination antiretroviral therapy; CYP, cytochrome P450; CXCL, CXC chemokine ligand; ddPCR, droplet digital polymerase chain reaction; EndoCAb, endogenous endotoxin-core antibody; GSDMD, gasdermin D; GLUT1, glucose transporter 1; HLA, human leukocyte antigen; HIV-1, human immunodeficiency virus type 1; HR, hazard ratio; hs-CRP, high-sensitivity C-reactive protein; IFN, interferon; IFNγ, interferon-gamma; IL, interleukin; IL-1β, interleukin-1 beta; I-FABP, intestinal fatty acid-binding protein; IL-2, interleukin-2; IL-4, interleukin-4; IL-6, interleukin-6; IL-7, interleukin-7; IL-15, interleukin-15; IL-18, interleukin-18; IL-18BPa, interleukin-18 binding protein A; IL-19, interleukin-19; INR, immunological non-response; IP-10, interferon gamma-induced protein 10; IR, immunological response; IS, indoxyl sulfate; KASP-PCR, Kompetitive Allele-Specific PCR; KIR, killer cell immunoglobulin-like receptor; LBP, lipopolysaccharide-binding protein; LC-MS, liquid chromatography-mass spectrometry; LPS, lipopolysaccharide; MALDI-TOF MS, matrix-assisted laser desorption/ionization time-of-flight mass spectrometry; MC, myristoylcarnitine; MCP, monocyte chemoattractant protein; MCP-1, monocyte chemoattractant protein-1; NK, natural killer; OR, odds ratio; OXPHOS, oxidative phosphorylation; PBMC, peripheral blood mononuclear cell; PC, palmitoylcarnitine; PCS, p-cresol sulfate; PCR-SSO, polymerase chain reaction sequence-specific oligonucleotide; PCR-SSP, polymerase chain reaction with sequence-specific primers; PGC1α, peroxisome proliferator-activated receptor-γ coactivator 1α; PTH, parathyroid hormone; RBP, retinol-binding protein; RBP4, retinol-binding protein 4; RTE, recent thymic emigrant; RT-qPCR, reverse transcription quantitative polymerase chain reaction; RR, relative risk; SC, stearoylcarnitine; SDF, stromal cell-derived factor; SDF-1, stromal cell-derived factor 1; SLC2A1, solute carrier family 2 member 1; SNP, single-nucleotide polymorphism; sCD14, soluble cluster of differentiation 14; TREC, T-cell receptor excision circle; TFAM, mitochondrial transcription factor A; ZAG, zinc-alpha-2-glycoprotein.

#### Genetic markers associated with INR.

**HLA alleles:** Pereira et al. [12] evaluated HLA associations with post-ART treatment nonresponse, defined as a CD4+ T-cell count <500 cells/µL after 12 months of ART, irrespective of viral load. The overall HLA-B allelic profile was statistically associated with treatment nonresponse (p = 0.0449). Among the alleles contributing most strongly to this association, HLA-B35 and HLA-B39 were associated with higher odds of treatment nonresponse [HLA-B35: OR 1.86, 95% CI 1.08–3.18; HLA-B39: OR 3.33, 95% CI 1.33–8.69]. HLA-B13 showed the largest point estimate (OR 4.06), but the estimate was imprecise, with a wide 95% CI that included the null value (0.45–36.62) [12]. In contrast, El-Beeli et al. [13] specifically evaluated immunological response among individuals with complete virological suppression (viral load ≤50 copies/mL). HLA-B15 was significantly associated with poor immunological response [RR 2.59, 95% CI 1.55–4.33; p = 0.023], while HLA-A68 was also significantly associated with poor immunological response [RR 2.37, 95% CI 1.37–4.13; p = 0.025] [13].

**KIR-related markers:** Soria et al. [14] evaluated multiple killer cell immunoglobulin-like receptor (KIR) genes and their HLA ligands. KIR2DL3, an inhibitory KIR involved in NK-cell regulation, was independently associated with a reduced risk of INR after adjustment for nadir CD4⁺ T-cell count (adjusted OR = 0.30, 95% CI: 0.12–0.74; p = 0.009). In contrast, KIR2DS2 was more frequent in the INR group than in the IR group in the unadjusted analysis (74.0% vs 54.8%; crude OR = 2.35, 95% CI: 1.06–5.27; p = 0.03), although the association was no longer statistically significant after adjustment. Several KIR–HLA combinations were also associated with INR: HLA-C1⁺/KIR2DL3⁺ and HLA-C1⁺/KIR2DS2^−^/KIR2DL3⁺ were associated with a lower risk of INR, whereas KIR2DS1⁺/C2⁺ was associated with a higher risk; detailed effect estimates are presented in Table 3.

**Cytokine and chemokine polymorphisms:** The rs1493013 polymorphism in IL-15, a cytokine essential for T-cell homeostasis, exhibited a trend toward association with INR (OR = 1.55, 95% CI: 0.99–2.43) [64]. Additionally, rs2430561 in IFNγ was significantly associated with INR (OR = 1.83, 95% CI: 1.10–3.05) [64], reinforcing the role of inflammatory responses in suboptimal CD4^+^ T-cell restoration.

**Chemokine receptor polymorphisms:** The CCR2 rs1799764 AG genotype showed a trend toward association with INR (OR = 1.50, *p* = 0.10) [15], aligning with prior findings implicating CCR2 in monocyte and T-cell trafficking. Furthermore, rs1801157 in CXCL12 demonstrated a potential association with INR status (*p* = 0.05) [15], emphasizing the impact of chemokine signaling in T-cell homeostasis and mobilization.

#### Protein and metabolomic markers in INR.

**Glucose metabolism:** The rs1385129 polymorphism in SLC2A1 (Glut1) was significantly associated with higher CD4^+^Glut1+ T cell percentages in individuals with INR (*p* = 0.038), with the GG genotype conferring a 4.67-fold increased risk of poor immune reconstitution (*p* = 0.04) [75]. Similarly, the rs1130214 polymorphism in AKT1 correlated with persistently elevated glycolytic activity in CD4^+^ T cells (*p* = 0.0068) [75], suggesting that excessive immune activation and metabolic stress contribute to suboptimal CD4^+^ T-cell recovery despite viral suppression.

**Cytokine and pyroptosis markers:** IL-18 (*p* = 0.001) [19] and caspase-1 (*p* = 0.046) [18] were significantly elevated in individuals with INR, indicating a heightened inflammatory state. IL-1β, while not significantly different (*p* = 0.370), exhibited a trend toward increased levels in individuals with INR [19].

**Adipokines and immune modulators:** Apelin and its receptor APLNR were significantly lower in individuals with INR (*p* = 0.011 and *p* = 0.007, respectively) [16], suggesting a role in immune homeostasis. Similarly, zinc alpha-2 glycoprotein (ZAG) (*p* = 0.040) [16] and retinol-binding protein 4 (RBP4) (*p* < 0.001) [16] were significantly decreased, implicating their role in metabolic-immune interactions.

### Other immunological markers

**Thymic output and TREC levels:** T-cell receptor excision circles (TREC) were significantly lower in individuals with INR (*p* < 0.01) [39], underscoring the diminished contribution of thymic-derived naïve T cells to immune reconstitution. Logistic regression models incorporating thymic function markers achieved a predictive accuracy of 87.1% (AUC = 0.908, *p* < 0.0001), highlighting the potential for TREC quantification as a prognostic tool for INR identification.

**CD4/CD8 ratio and CD8 ⁺ T-cell phenotype:** Vos et al. [47] reported that lower pre-cART and most recently measured CD4/CD8 ratios were statistically significantly associated with INR in the discovery cohort (both p < 0.001), although these associations were not significantly replicated in the validation cohort. Immunophenotyping further showed reduced absolute numbers of CD8⁺ naïve T cells, CD8 TC17 cells, and CD8 TC1/17 cells in the INR group. Increased proportions of HLA-DR⁺ and CD38⁺ CD8⁺ T-cell subpopulations were also observed in the discovery cohort; however, these activation-marker patterns were not consistently reproduced in the validation cohort.

**NK-cell immunophenotype:** Vos et al. [47] performed extensive immunophenotyping of innate and adaptive immune-cell populations in independent discovery and validation cohorts. Absolute NK-cell and NK T-like-cell numbers were lower in the INR group than in the IR group, whereas the overall proportions of NK cells did not differ between groups. The INR group also showed increased HLA-DR⁺ subpopulations of NK cells and NK T-like cells, with a similar direction of change in the validation cohort. In contrast, alterations in CD38⁺ NK-cell subsets were not consistent between the discovery and validation cohorts.

**Recent thymic emigrants (RTE):** RTE percentages were significantly reduced in the INR group compared with the IR group (p = 0.038) [32], indicating impaired thymic output as a key driver of poor CD4+ T-cell recovery.

**Microbial translocation:** Several markers related to microbial translocation were evaluated in relation to INR. LPS, LBP, sCD14, and EndoCAb differed significantly between the INR and IR groups in individual studies, whereas I-FABP did not show a significant between-group difference [33,46]. Two gut microbial metabolites, p-cresol sulfate (PCS) and indoxyl sulfate (IS), were also elevated in the INR group [73,86].

### Publication bias

Publication bias was assessed using funnel plots for four meta-analytic domains with relatively large numbers of contributing studies: sex, age, transmission route, and co-infection. These plots are shown in S1–S3 and S5 Figs in S1 File. The funnel plots appeared approximately symmetrical, with no clear visual evidence of publication bias. Egger’s tests likewise showed no statistically significant evidence of small-study effects (all p > 0.05).

## Discussion

This systematic review and meta-analysis synthesized data from multiple studies to identify predictors of INR in PLWH undergoing ART, spanning demographic, clinical, and molecular domains. To the best of our knowledge, this is the first comprehensive review to integrate evidence across all four predictor categories in the context of INR. Our analysis reveals that INR is a multifactorial condition driven by a convergence of host-related variables and biological dysregulation. We observed a modest pooled association between male sex and INR, whereas older age, low baseline CD4+ T-cell count, and advanced HIV stage at ART initiation were also associated with poorer immune recovery. Additional exploratory analyses indicated that the observed association with sex should be interpreted in the context of differences in parenteral transmission and viral co-infection burden across study populations. Clinical factors associated with INR included hepatitis B and C co-infections, tuberculosis, and advanced HIV stage, whereas underweight status was not significantly associated with INR. On a molecular level, our findings highlight the roles of genetic polymorphisms, inflammatory cytokines, and metabolic disturbances associated with INR, underscoring the multifactorial nature of impaired immune reconstitution. These findings highlight potentially modifiable contributors to impaired immune recovery and biological pathways, particularly chronic immune activation and impaired T-cell homeostasis, that may represent targets for future therapeutic investigation.

### Demographic factors associated with INR

The pooled analysis showed an association between male sex and INR (OR 1.33, 95% CI 1.12–1.58), accompanied by substantial between-study heterogeneity (I² = 66.7%). This association therefore warrants cautious interpretation and should not automatically be attributed to biological sex alone. In our exploratory meta-regression, higher study-level IDU prevalence was significantly associated with a smaller male-versus-female effect estimate, suggesting that differences in parenteral HIV transmission may contribute to the observed sex association. Higher HCV prevalence showed a similar direction of association, although the moderator effect was not statistically significant, whereas HBV prevalence did not explain variation in the sex effect. These findings partially support the hypothesis that epidemiological factors associated with HIV acquisition may contribute to the apparent association between male sex and impaired immune recovery. However, they do not indicate that the sex association can be entirely explained by IDU or viral hepatitis. Biological mechanisms related to sex differences in immune activation and hormonal regulation remain plausible; estrogen, particularly 17β-estradiol, has been reported to modulate antiviral and inflammatory immune responses [87,88]. Nevertheless, because our analyses were based on aggregate study-level characteristics, the independent contribution of biological sex cannot be distinguished conclusively from differences in transmission route and co-infection burden.

Advanced age is a well-established risk factor for INR, as older individuals initiating ART exhibit reduced thymic output and slower immune recovery [1,89]. Thymic involution, a natural aging process, leads to diminished production of naive T cells, which is crucial for immune reconstitution following ART initiation [90,91]. The compromised regenerative capacity of the thymus in older PLWH further exacerbates immune recovery deficits, making age a significant determinant of INR [92,93].

### Clinical factors associated with INR

Our findings regarding IDU and viral co-infections provide additional context for the observed association between sex and INR. HIV acquisition through injection drug use was associated with poorer immune recovery in the primary meta-analysis, consistent with previous reports linking IDU with suboptimal immune reconstitution [94,95]. The additional meta-regression showed that cohorts with a higher prevalence of IDU tended to have a smaller male-versus-female effect estimate. This finding suggests that differences in parenteral transmission patterns may partly contribute to the apparent association between sex and INR. Similarly, our data show that HCV co-infection is associated with up to a two-fold increased risk of INR. This aligns with prior reports indicating that HCV co-infection accelerates immune activation and enhances CD4^+^ T-cell depletion, complicating immune reconstitution despite ART [96,97]. Even after successful HCV treatment, residual inflammation and hepatic fibrosis may persist, further hindering CD4^+^ T-cell recovery [98]. The duration and temporal context of HIV infection and co-infections may also influence immunological recovery. Several included studies evaluated measures such as duration of HIV infection, time since diagnosis, or the interval between diagnosis and ART initiation, with some reporting associations between longer pre-ART disease duration and poorer immune recovery, whereas others found no independent association [3,42,47,61]. These heterogeneous findings likely reflect differences in how HIV duration was defined and measured, as well as differences in baseline immunodeficiency and timing of ART initiation. For HBV, HCV, and tuberculosis, the included studies generally reported the presence, activity, or timing of co-infection rather than its duration; therefore, the potential effect of prolonged co-infection exposure on immune recovery could not be directly assessed.

We also found that the advanced HIV clinical stage at ART initiation is strongly associated with INR. Late-stage disease is linked to persistent immune dysregulation and chronic inflammation [55,99]. This is consistent with previous studies indicating that individuals presenting with advanced disease often experience prolonged immune suppression, increased comorbidities, and heightened systemic inflammation, all of which contribute to poor CD4^+^ T-cell reconstitution [35,52]. Chronic immune activation remains a significant barrier to immune recovery in these individuals, as sustained inflammation promotes T-cell exhaustion and disrupts immune homeostasis [100].

### Mechanistic synthesis I: Chronic immune activation and inflammasome-driven pyroptosis

Chronic immune activation remains a key mechanism underlying immune recovery failure in individuals with INR despite effective ART, as demonstrated in Figs 2 and 3. Our review identified elevated levels of inflammatory cytokines, particularly IL-18 and caspase-1, as markers of ongoing immune activation [18,19]. Pyroptosis, a caspase-1-dependent form of inflammatory cell death, plays a central role in this process [101–104]. Triggered by the NLRP3 inflammasome, pyroptosis leads to GSDMD-mediated membrane pore formation and release of IL-1β and IL-18, amplifying inflammation and accelerating CD4⁺ T-cell depletion [101–104]. Recent findings indicate that the CARD8 inflammasome can sense HIV-1 through direct cleavage by the viral protease, triggering pyroptosis and the release of IL-1β and IL-18. Toll-like receptor signaling may modulate this response prior to infection, but the primary sensing mechanism in this context appears to be CARD8-mediated rather than canonical PRR pathways [105,106].

**Fig 2:**
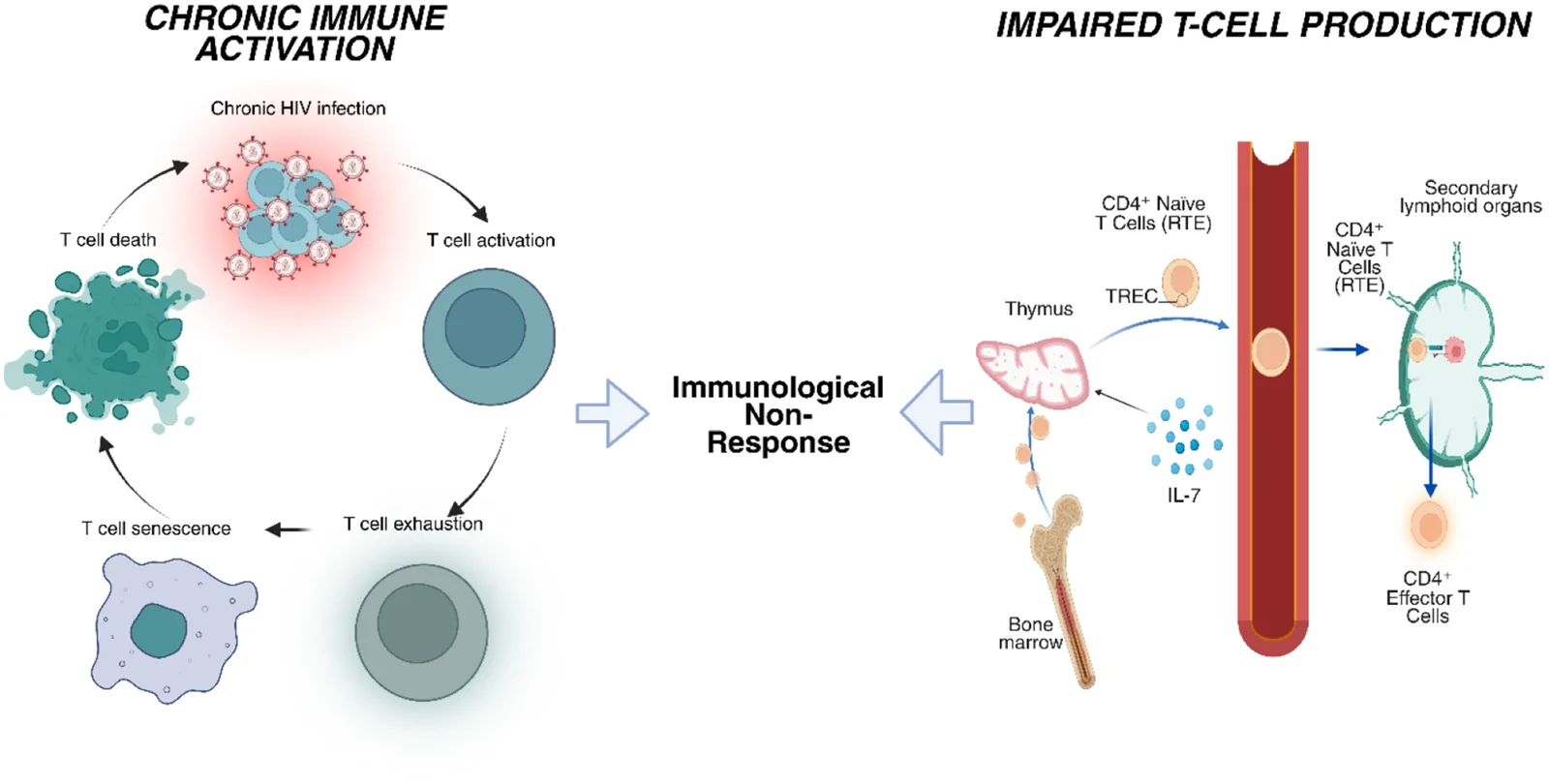
Potential mechanisms underlying immunological non-response in PLWH: interplay between chronic immune activation and impaired T-cell production. Chronic immune activation induces persistent T-cell stimulation, leading to exhaustion, senescence, and apoptosis. Concurrently, impaired T-cell production, marked by reduced IL-7 signaling, thymic output, and TREC levels, limits the generation of naïve and effector CD4 ⁺ T cells. Together, these processes disrupt CD4⁺ T-cell homeostasis despite viral suppression, contributing to the INR phenotype.

**Fig 3:**
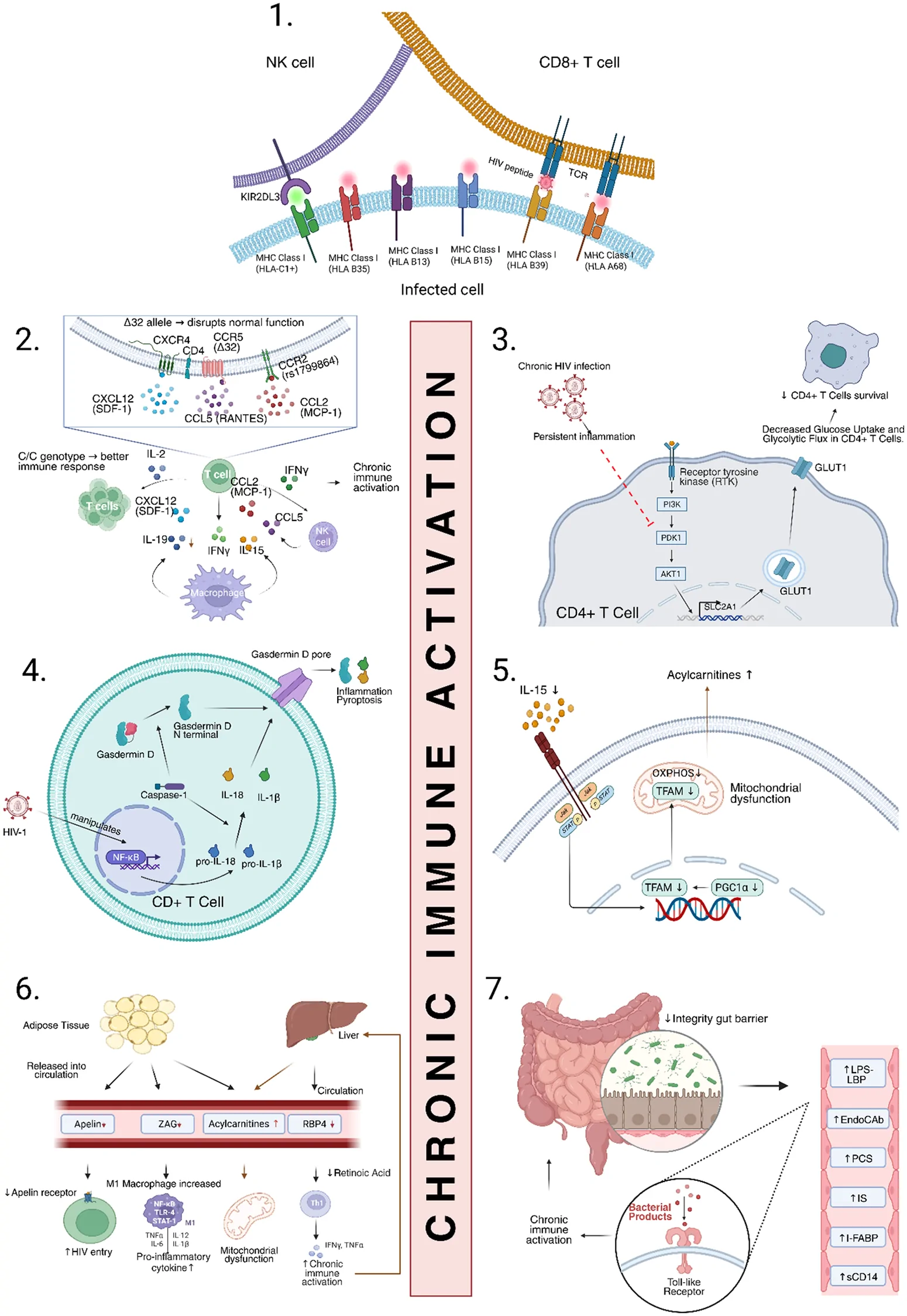
Multifactorial drivers of chronic immune activation leading to immunological non-response in people living with HIV (PLWH). 1. Genetic variants in HLA and KIR. HLA class I alleles (e.g., B13, B15, B35, B39, A68) influence CD8⁺ T cell responses via antigen presentation. KIR2DL3, through interaction with HLA-C1, modulates NK cell activity. The compound genotype HLA-C1⁺/KIR2DL3⁺ is linked to reduced INR risk, while other alleles increase susceptibility. 2. Chemokine signaling polymorphisms. Variants in CCR5 (Δ32), CCR2, CXCL12, and CCL5 disrupt immune cell trafficking, enhancing monocyte/macrophage recruitment and elevating IL-1β, IL-18, and IFN-γ levels, amplifying immune activation. 3. Metabolic reprogramming in CD4⁺ T cells. SLC2A1 and AKT polymorphisms increase GLUT1 expression and glycolytic activity, promoting CD4⁺ T cell dysfunction and impaired survival, key features of INR. 4. Inflammasome activation and pyroptosis. HIV triggers NF-κB-mediated inflammasome assembly and caspase-1 activation, cleaving gasdermin D and inducing pyroptosis. Elevated IL-1β and IL-18 serve as markers of this proinflammatory cascade. 5. Mitochondrial dysfunction. Reduced TFAM and PGC1α expression impairs mitochondrial biogenesis and OXPHOS. Accumulated acylcarnitines reflect oxidative stress and immune exhaustion. 6. Adipokines and hepatokines. Lower circulating apelin, ZAG, and RBP4 levels impair T cell homeostasis and anti-inflammatory regulation, contributing to systemic immune dysregulation. 7. Microbial translocation. Disruption of gut epithelial integrity leads to translocation of LPS, PCS, and IS, activating TLR pathways. Elevated sCD14, LBP, EndoCAb, and I-FABP indicate dysbiosis and persistent systemic inflammation.

Persistent activation of this inflammasome-cytokine axis in individuals with INR contributes to systemic inflammation and impaired immune reconstitution, even under viral suppression. Additionally, gut barrier disruption promotes microbial translocation, further driving inflammasome activation and maintaining a pro-inflammatory environment [107–109]. These findings highlight a self-perpetuating cycle of pyroptosis, cytokine release, and immune cell loss as central to INR pathology, underscoring potential targets for adjunctive therapeutic intervention.

### Host genetics and immunogenetics associated with INR

In addition to the inflammasome-mediated pyroptotic pathways described earlier, our review underscores the critical role of host genetic variation, particularly within genes encoding chemokine receptors and their ligands, in sustaining immune dysregulation among individuals with INR. These genetic polymorphisms can influence cytokine signaling, immune cell trafficking, and T-cell homeostasis, thereby modulating the immune reconstitution process even in effective ART. Specifically, we identified variants in CCR5 and CCR2 that have been linked to dysregulated inflammatory responses and reduced CD4⁺ T-cell recovery [15,17]. One of the most studied examples is the CCR5 ∆32 deletion, which is well known for conferring resistance to HIV infection in homozygous individuals for the mutation due to the absence of CCR5 expression on the cell surface. However, in heterozygous individuals, this variant may still influence the cytokine milieu and immune cell migration, potentially affecting immune restoration dynamics [110,111]. Likewise, the rs1799864 polymorphism in CCR2 has been associated with altered MCP-1/CCL2 levels, which may influence monocyte recruitment and immune activation, potentially contributing to systemic inflammation in individuals with poor immune recovery [63,112].

Furthermore, our review identified the CCL5/CCR5 axis dysregulation as a key mechanism contributing to INR pathogenesis. Specifically, we found that the rs2280788 variant in CCL5 is frequently associated with elevated RANTES (Regulated on Activation, Normal T Cell Expressed and Secreted) expression, a chemokine that facilitates CD8⁺ T-cell recruitment [63]. While RANTES contributes to antiviral defense by attracting CD8+ T cells to sites of infection, excessive expression leads to chronic immune activation and T-cell exhaustion, impairing immune function [113]. Overexpression of CCL5 correlates with increased immune activation and ineffective immune recovery in PLWH [114]. Furthermore, prolonged immune activation driven by high RANTES levels disrupts CD4^+^ T-cell differentiation, favoring an ineffective, exhausted phenotype in viral clearance [115]. This paradoxical effect highlights the role of chemokine dysregulation in the persistence of INR, where an exaggerated immune response ultimately contributes to immunosuppression and disease progression.

Our review also identified the rs1801157 variant in CXCL12, which encodes stromal cell-derived factor 1 (SDF-1), as being associated with poor immune recovery in individuals with INR [63]. Individuals carrying the T allele demonstrated reduced SDF-1 expression, which may impair hematopoietic progenitor migration and limit CD4⁺ T-cell regeneration [63]. This impaired recovery appears to be further aggravated by systemic inflammation [63]. Low SDF-1 expression in this condition has been linked to persistent systemic inflammation, with increased levels of IL-6 and TNF-α, further exacerbating immune dysfunction and hindering immune reconstitution following cART initiation [116]. The chronic inflammatory state perpetuated by chemokine dysregulation creates a feedback loop where immune activation fails to resolve, leading to ongoing tissue damage and defective T-cell restoration in individuals with INR.

Our review identified several HLA class I variants potentially associated with suboptimal immune recovery during ART. Among virologically suppressed individuals, El-Beeli et al. [13] reported significant associations of HLA-A68 and HLA-B15 with poor immunological response. In contrast, Pereira et al. [12] evaluated a broader post-ART treatment-nonresponse phenotype that did not require virological suppression and identified an overall association between the HLA-B allelic profile and treatment nonresponse. HLA-B35 and HLA-B39 showed elevated allele-specific odds of treatment nonresponse, whereas HLA-B13 showed the largest point estimate but with substantial imprecision, as its 95% confidence interval included the null value. These findings suggest a potential role of HLA variation in post-ART immune recovery; however, differences in virological status and operational definitions of immunological response across studies limit direct comparison. Mechanistically, previous studies suggest that selected HLA class I alleles may influence HIV-related immune recovery through differences in antiviral T-cell responses, immune activation, and T-cell exhaustion [117,118]. The potential impact of these HLA alleles is further supported by evidence that HLA-B35 and HLA-B39 are associated with suboptimal antiviral CD8⁺ T-cell responses. Studies have shown that CD8⁺ T cells restricted by these alleles exhibit functional exhaustion, characterized by increased expression of inhibitory receptors such as CTLA-4 and PD-1 and reduced cytolytic activity [119,120]. Consistent with the relevance of the CD8⁺ T-cell compartment, Vos et al. [47] found that lower pre-cART and most recently measured CD4/CD8 ratios were associated with INR in the discovery cohort, although these associations were not significantly replicated in the validation cohort. The same study also demonstrated reductions in several CD8⁺ T-cell subsets, including naïve, TC17, and TC1/17 populations, together with alterations in CD8⁺ T-cell activation markers. These findings suggest that incomplete immune recovery involves not only insufficient CD4⁺ T-cell restoration but also persistent perturbations of CD4/CD8 T-cell homeostasis. Additionally, HLA-A68 has been associated with poor HIV control, including higher viral load and lower CD4 counts, suggesting a potential role in suboptimal immune recovery. Although the precise mechanisms remain elusive, this allele may influence immune regulation [121]. While our review highlights HLA-B39 as a potential risk allele for poor immune recovery under ART, other studies have reported a protective association of this allele in ART-naïve cohorts [122]. For instance, HLA-B39 has been identified as a protective factor among Spanish long-term non-progressors (LTNPs), suggesting a role in delayed disease progression in the absence of ART [122]. This discrepancy underscores the context-dependent nature of HLA effects, which may vary across populations and clinical scenarios. Indeed, geographic and demographic variability has been documented, with studies from Argentine and Indian populations reporting HLA-B39 as a risk allele [117], suggesting that host genetic background significantly modulates the immunological consequences of HLA class I polymorphisms.

Beyond the effects of individual HLA class I alleles, interactions between HLA class I molecules and killer cell immunoglobulin-like receptors (KIRs) may further influence antiviral immunity and immune recovery [123,124]. Our review identified KIR2DL3, an inhibitory KIR involved in NK-cell regulation, as being independently associated with a reduced risk of immunological non-response (INR) after adjustment for nadir CD4⁺ T-cell count [14]. In contrast, the activating KIR2DS2 was more frequent in the INR group than in the IR group in the unadjusted analysis, although this association was attenuated and no longer statistically significant after adjustment [14]. Importantly, the findings of Soria et al. also indicate that KIR–HLA combinations may be relevant to immune recovery: HLA-C1⁺/KIR2DL3⁺ and HLA-C1⁺/KIR2DS2^−^/KIR2DL3⁺ were associated with a reduced risk of INR, whereas KIR2DS1⁺/C2⁺ was associated with an increased risk [14]. These observations suggest that the balance between activating and inhibitory KIR signaling, together with the corresponding HLA ligands, may be more informative than individual KIR genotypes alone. Beyond these immunogenetic associations, direct immunophenotyping by Vos et al. [47] demonstrated lower absolute NK-cell and NK T-like-cell numbers in the INR group, despite comparable overall NK-cell proportions, together with increased HLA-DR⁺ NK-cell and NK T-like-cell subpopulations. However, changes in CD38⁺ NK-cell subsets were not consistently reproduced across the discovery and validation cohorts, indicating that NK-cell alterations in INR may involve specific changes in cell abundance and activation phenotype rather than uniform global NK-cell activation.

Furthermore, our review highlights that the compound genotype HLA-C1⁺/KIR2DL3⁺ may be associated with a lower risk of poor immune recovery [14]. This epistatic interaction underscores how KIR-HLA co-expression impacts NK cell-mediated control of HIV and subsequent immune restoration [123,125]. The functional pairing of KIR2DL3 with its cognate ligand, HLA-C1, appears to facilitate a balanced immunoregulatory environment, supporting efficient NK cell activation in response to infected cells while minimizing unnecessary immune activation that can lead to tissue damage or immune exhaustion [126].

### Immunometabolic and proteomic signatures associated with INR

Mitochondrial dysfunction plays a crucial role in the pathogenesis of immune recovery failure in HIV-infected individuals, significantly impacting T-cell proliferation and overall immune competency. Our review identified the downregulation of two critical regulators of mitochondrial biogenesis, peroxisome proliferator-activated receptor gamma coactivator 1-alpha (PGC1α) and mitochondrial transcription factor A (TFAM), as consistent findings in individuals with INR [66]. These regulators are essential for maintaining mitochondrial function and supporting the energy demands of proliferating T cells. In parallel, previous studies have suggested that interleukin-15 (IL-15), a cytokine involved in T-cell survival and proliferation, is reduced in some HIV-infected patients [127]. Although IL-15 was not identified as a dysregulated output in our review, experimental in vitro studies have shown that IL-15 exposure can restore the expression of PGC1α and TFAM, thereby enhancing mitochondrial fitness in regulatory T cells from individuals with INR [128]. The downregulation of PGC1α and TFAM contributes to compromised oxidative phosphorylation, diminished ATP production, and heightened oxidative stress in CD4⁺ and CD8⁺ T cells. Although an increase in mitochondrial mass has been reported in HIV-infected individuals, mitochondrial quality and bioenergetic function remain severely impaired [112]. This dysfunction leads to excessive production of reactive oxygen species (ROS), resulting in mitochondrial DNA damage, cellular senescence, and apoptosis [129], all hindering immune restoration.

In addition to mitochondrial dysfunction, our review underscores the presence of metabolic disturbances in HIV-infected individuals, as evidenced by the accumulation of acylcarnitines, intermediates in fatty acid metabolism. Elevated acylcarnitine levels indicate an imbalance in mitochondrial fatty acid oxidation, contributing to systemic metabolic stress and increased susceptibility to apoptosis [130]. The excessive accumulation of these metabolites is a hallmark of mitochondrial dysfunction and has been linked to chronic inflammation, as acylcarnitines can promote inflammatory pathways that drive immune exhaustion [116]. This metabolic maladaptation, characterized by an increased reliance on glycolytic pathways due to impaired mitochondrial respiration, results in sustained inflammation and poor immune reconstitution, even in patients receiving ART [131]. Studies have also found that acylcarnitine accumulation is not exclusive to HIV infection but is observed in other inflammatory conditions, such as septic shock and cardiovascular diseases, reinforcing the role of metabolic dysregulation in immune dysfunction [132].

Additionally, we identified dysregulation of the apelin signaling pathway as a potential axis of immune impairment in individuals with INR [16]. Apelin, a bioactive peptide with established anti-inflammatory properties, functions through its receptor APLNR to regulate macrophage activation and immune modulation [133,134]. While previous studies have demonstrated the immunosuppressive effects of apelin, particularly its ability to downregulate pro-inflammatory cytokines such as TNF-α and IL-1β [135], our review identified reduced expression of the apelin receptor (APLNR) in individuals with INR as a consistent finding [16]. This reduction may compromise apelin-mediated signaling, leading to heightened macrophage activation and sustained cytokine production.

Consistent with our findings, prior studies have demonstrated that decreased levels of adipokines, particularly RBP4 and ZAG, are associated with heightened systemic inflammation and poor immune recovery in individuals with INR. RBP4 deficiency promotes macrophage polarization toward the pro-inflammatory M1 phenotype, enhancing immune activation and disrupting metabolic balance [136,137]. Similarly, reduced expression of ZAG, a glycoprotein secreted by adipose tissue with known roles in lipid metabolism and immune regulation, has been observed in chronic inflammatory states common among individuals with INR [16,138,139]. Mechanistically, ZAG suppresses pro-inflammatory cytokines such as TNF-α and IL-6 while activating anti-inflammatory pathways, including p-ERK and TGF-β1, which are essential for endothelial homeostasis and immune modulation [138–140]. The downregulation of these adipokines reflects a disrupted adipose-immune axis, potentially perpetuating systemic inflammation and impairing CD4⁺ T-cell recovery in HIV-infected individuals with INR.

At the genetic level, our review identified the rs1385129 variant in the SLC2A1 gene, which encodes glucose transporter-1 (GLUT-1), as a significant contributor to immune recovery failure in individuals with INR. Individuals with the Guanine-Guanine (GG) genotype demonstrated both elevated GLUT-1 expression on CD4⁺ T cells and a 4.67-fold higher risk of INR compared with Guanine-Adenine or Adenine-Adenine (GA/AA) carriers [75]. This genotype promotes a metabolic shift toward aerobic glycolysis, a hallmark of chronically activated and exhausted T cells, and is strongly linked to increased glucose uptake and immune dysfunction [141,142]. These findings highlight the contribution of GLUT-1 dysregulation, alongside previously discussed metabolic abnormalities, as a key determinant of the INR phenotype despite effective ART.

Broader metabolic dysregulation was also evident in the form of increased glucose metabolism and Nicotinamide Adenine Dinucleotide (NAD⁺) depletion in HIV-infected individuals with INR status. Upregulation of glucose transporter 1 (GLUT-1) and elevated CD38 activity have been linked to enhanced glycolysis, mitochondrial dysfunction, and immune exhaustion [141–143]. These bioenergetic disturbances promote immune senescence and may prevent CD4⁺ T-cell regeneration, even under ART [144,145]. Our review also identified key genetic polymorphisms in glucose metabolism pathways as significant contributors to INR in HIV-infected individuals. Notably, we found that the rs1385129 variant in the SLC2A1 gene (encoding GLUT1) and the rs1130214 variant in AKT1 are associated with increased GLUT1 expression on CD4⁺ T cells, heightened glucose uptake, and persistently elevated glycolytic activity [75]. Specifically, individuals carrying the GG genotype of rs1385129 demonstrated a higher proportion of GLUT1⁺ CD4⁺ T cells and exhibited a significantly increased risk of poor immune recovery despite ART [75]. Although the rs1130214 variant in AKT1 was associated with elevated glycolytic activity, it did not reach statistical significance in clinical outcomes [75]. Nevertheless, our findings align with broader evidence that GLUT1 dysregulation and sustained glucose-driven immune activation are prominent features in HIV-infected individuals [141,142].

This dysregulated metabolic state appears to be sustained by inflammatory signaling, particularly through the PI3K-AKT-mTORC1 axis [146,147]. Chronic HIV-related inflammation has been shown to upregulate GLUT1 expression and shift immune cells, particularly CD4⁺ T cells and monocytes, towards aerobic glycolysis, a metabolic phenotype commonly associated with immune activation and exhaustion [148,149]. Despite increased glucose transporter expression, T cells in individuals with INR remain functionally compromised, as glucose uptake and mitochondrial respiration remain inadequate to support proliferation and effector function [150]. Persistent HIV-related inflammation and metabolic reprogramming, characterized by increased GLUT1 expression and impaired mitochondrial function, may limit the metabolic flexibility of immune cells, particularly T cells, thereby disrupting their capacity to meet energy demands during activation [71,151,152]. Our review adds to this framework by highlighting that these genetic and molecular alterations converge to maintain a state of metabolic exhaustion that obstructs CD4⁺ T-cell recovery in the INR phenotype.

### Mechanistic synthesis II: Impaired thymopoiesis and IL-7/IL-7R axis dysfunction

In addition to chronic immune activation being a central mechanism of INR, our review underscores the contribution of insufficient CD4⁺ T-cell production, driven by impaired thymic function, in limiting immune recovery during ART. Thymopoiesis within the thymus is essential for generating naïve T cells, especially in individuals presenting with marked immunodeficiency at the onset of therapy, as illustrated in Fig 2. In individuals with INR, two established markers of thymic activity, T-cell receptor excision circles (TREC) and recent thymic emigrants (RTEs), were consistently found to be reduced [32,39]. Specifically, studies reported lower levels of signal-joint TRECs in individuals with INR throughout ART, suggesting sustained impairment in thymic output [39]. Likewise, the proportion of RTEs was significantly diminished in the INR group compared with the IR group, reflecting the limited influx of newly generated naïve T cells [32].

This deficit in thymic activity is exacerbated by chronic immune activation and persistent viral replication, which drive CD8⁺ T-cell hyperactivation, systemic inflammation, and continued CD4⁺ T-cell depletion [153]. The ensuing inflammatory milieu creates an unfavorable thymic environment, leading to structural and functional thymic deterioration [154–156]. Moreover, the persistence of HIV within the thymic niche may impair thymopoiesis by disrupting cytokine balance, particularly via type I interferons and pro-inflammatory mediators, which alters essential signaling pathways for T-cell development and compromise the integrity and function of thymic stromal cells [157,158]. Supporting evidence for interferon-driven thymic impairment is also observed in chronic viral infection models such as lymphocytic choriomeningitis virus (LCMV), which, although not HIV-specific, exhibit mechanistic parallels relevant to inflammation-induced thymic dysfunction [157,158]. These combined processes, immune-driven inflammation and direct viral interference are consistent with broader evidence indicating that HIV-associated thymic damage restricts immune regeneration [154,159,160].

Our review further highlights the essential role of interleukin-7 (IL-7) in thymopoiesis and peripheral T-cell homeostasis through CD127-mediated signaling, which promotes T-cell survival, proliferation, and STAT5-driven CD25 expression [77]. Despite elevated IL-7 levels often observed in lymphopenic individuals with INR, effective T-cell expansion remains limited, suggesting a functional impairment in the IL-7/IL-7R axis. This paradox may be explained by reduced IL-7 receptor expression or disrupted downstream signaling, as evidenced by the poor correlation between CD127 levels and IL-7-induced T-cell proliferation [161–164]. These findings underscore that receptor presence alone does not guarantee signaling competence, potentially limiting thymic and peripheral immune reconstitution in individuals with INR.

Despite the comprehensive findings of this systematic review and meta-analysis, we acknowledge several important limitations. First, statistical heterogeneity was observed in several pooled estimates, particularly for demographic and clinical predictors such as age, transmission route, and baseline CD4⁺ count. This heterogeneity likely reflects variations in study populations, definitions of immunological non-response, ART regimens, and regional differences in healthcare systems. However, the observed heterogeneity does not necessarily compromise the validity of the findings. To account for this, we employed a random-effects model in most meta-analyses, which provides more conservative and generalizable estimates by incorporating both within-study and between-study variability. Moreover, the direction and significance of most associations remained consistent across studies, suggesting that the observed effects are robust despite population-level differences. The exploratory analyses examining sex, IDU, and viral co-infections also require cautious interpretation. These meta-regressions used aggregate study-level prevalence rather than individual-participant data and therefore cannot establish participant-level confounding, mediation, or statistical interaction between sex, IDU, and co-infection status. In particular, associations observed between study-level IDU prevalence and the magnitude of the sex effect are susceptible to ecological bias. Furthermore, moderator data were available for only 10 studies for IDU, 14 for HCV, and 13 for HBV, which limited statistical power and precluded a reliable multivariable meta-regression incorporating all three moderators simultaneously. The primary studies also did not provide sufficient sex-by-INR data within IDU or viral co-infection strata to permit pooled stratified analyses. Therefore, an independent biological effect of sex cannot be established from the available aggregate evidence. The temporal characteristics of HIV infection and co-infections were also incompletely and inconsistently reported across the primary studies. Measures of HIV duration varied substantially, including time since diagnosis, time to ART initiation, and duration of advanced immunosuppression, precluding meaningful quantitative pooling. Moreover, none of the evaluated primary studies provided directly usable data on the duration of HBV, HCV, or tuberculosis co-infection. These unmeasured temporal factors may therefore contribute to residual confounding and between-study heterogeneity in the observed associations with INR. Oncological comorbidities and anticancer treatments represent another potential source of residual confounding. Among all included studies, only three explicitly excluded cancer history, baseline malignancy, or chemotherapy/immunosuppressive treatment; three accounted for malignancy analytically or through sensitivity analyses; and four reported malignancy or chemotherapy exposure without excluding affected participants. In the remaining studies, baseline oncological comorbidity and treatment status were not consistently reported. Because malignancy and anticancer therapies may independently contribute to lymphopenia and CD4⁺ T-cell depletion, their incompletely reported distribution across INR and IR groups may have introduced residual confounding into some of the observed associations. Second, there were substantial inconsistencies in the definition, assessment, and threshold values of predictive factors across studies. This was particularly evident among molecular, genetic, and immunological markers, where variations in assay methods and reporting standards further contributed to heterogeneity. Third, for many predictors included in the meta-analysis, the number of eligible studies was fewer than ten, which limits statistical power, reduces the robustness of effect estimates, and hinders the reliability of subgroup or sensitivity analyses. Fourth, the absence of a standardized definition of INR represents an important limitation. The included studies used heterogeneous criteria based on different absolute CD4⁺ T-cell thresholds, magnitudes of CD4⁺ T-cell recovery, and durations of ART or virological suppression. Consequently, participants classified as INR may not represent entirely comparable populations across studies, potentially contributing to between-study heterogeneity and limiting the generalizability of the pooled estimates. Nevertheless, this review contributes by integrating diverse demographic, clinical, and molecular determinants, offering a more comprehensive framework to understand the multifactorial mechanisms underlying INR in PLWH.

## Conclusion

This systematic review and meta-analysis shows that immunological non-response (INR) among people living with HIV receiving antiretroviral therapy is influenced by a complex combination of demographic, clinical, and biological factors. Male sex showed a modest pooled association with INR, whereas older age, intravenous drug use, viral co-infections, and low baseline or nadir CD4⁺ T-cell counts were also associated with impaired immune recovery. The association with male sex should be interpreted cautiously, as differences in parenteral transmission patterns, particularly the prevalence of IDU, may partly contribute to the observed relationship. In addition, emerging evidence on genetic, metabolic, and immunological biomarkers provides further insight into mechanisms that may limit immune reconstitution despite successful viral suppression.

These findings highlight the importance of a more individualized approach to HIV treatment, where conventional clinical indicators are complemented by molecular and immunological profiling to identify patients at higher risk early in therapy. Future research should prioritize longitudinal studies examining changes in inflammatory, metabolic, and thymic function markers during ART, as well as experimental trials of adjunctive therapies such as IL-7 supplementation or inflammasome modulation. Developing practical risk prediction models that integrate demographic, clinical, and biomarker data may also improve early detection and support more effective strategies for sustained immune recovery. Finally, the lack of a universally accepted definition of INR remains an important challenge, highlighting the need for standardized criteria to improve comparability across future studies.

## Supporting information

S1 File Supporting information. PRISMA checklist, search strategies, supplementary figures (S1–S9 Figs), and supplementary tables (S1–S4 Tables).
(DOCX)

S1 Data Extracted dataset for meta-analysis. (XLSX)
