# Supplementary material for: Predictors of immunological non-response despite virological suppression in people living with HIV on antiretroviral therapy: A systematic review and meta-analysis
Source: PLoS One. 2026 Oct 12;21(10):e0358793. doi: 10.1371/journal.pone.0358793 (PMC13657295; doi:10.1371/journal.pone.0358793)
Supplement: Supplementary file 1 [file pone.0358793.s001.docx]

Supporting Information

**Predictors of immunological non-response despite virological suppression in people living with HIV on antiretroviral therapy: a systematic review and meta-analysis**

Authors:

Brian Eka Rachman

Yehuda Tri Nugroho Supranoto

Fitri Revita Nurannisa

Siti Mei Saroh

Siti Qamariyah Khairunisa

Citrawati Dyah Kencono Wungu

Dian Paramita Kartikasari

Nasronudin Nasronudin

Masanori Kameoka

Muhammad Miftahussurur

Retno Pudji Rahayu

Afif Nurul Hidayati

# S1 File. PRISMA Checklist

| **Section and Topic** | **Item #** | **Checklist item** | **Location where item**  **is reported** |
| --- | --- | --- | --- |
| **TITLE** | | |  |
| Title | 1 | Identify the report as a systematic review. | Page 1 |
| **ABSTRACT** | | |  |
| Abstract | 2 | See the PRISMA 2020 for Abstracts checklist. | Page 3 |
| **INTRODUCTION** | | |  |
| Rationale | 3 | Describe the rationale for the review in the context of existing knowledge. | Page 4-5 |
| Objectives | 4 | Provide an explicit statement of the objective(s) or question(s) the review addresses. | Page 5-6 |
| **METHODS** | | |  |
| Eligibility criteria | 5 | Specify the inclusion and exclusion criteria for the review and how studies were grouped for the syntheses. | Page 6 |
| Information sources | 6 | Specify all databases, registers, websites, organisations, reference lists and other sources searched or consulted to identify studies. Specify the date when each source was last searched or consulted. | Page 7 |
| Search strategy | 7 | Present the full search strategies for all databases, registers and websites, including any filters and limits used. | Page 7 |
| Selection process | 8 | Specify the methods used to decide whether a study met the inclusion criteria of the review, including how many reviewers screened each record and each report retrieved, whether they worked independently, and if applicable, details of automation tools used in the process. | Page 6-8 |
| Data collection process | 9 | Specify the methods used to collect data from reports, including how many reviewers collected data from each report, whether they worked independently, any processes for obtaining or confirming data from study investigators, and if applicable, details of automation tools used in the process. | Page 7 |
| Data items | 10a | List and define all outcomes for which data were sought. Specify whether all results that were compatible with each outcome domain in each study were sought (e.g. for all measures, time points, analyses), and if not, the methods used to decide which results to collect. | Page 8 |
|  | 10b | List and define all other variables for which data were sought (e.g. participant and intervention characteristics, funding sources). Describe any assumptions made about any missing or unclear information. | Page 8 |
| Study risk of bias assessment | 11 | Specify the methods used to assess risk of bias in the included studies, including details of the tool(s) used, how many reviewers assessed each study and whether they worked independently, and if applicable, details of automation tools used in the process. | Page 9 |
| Effect measures | 12 | Specify for each outcome the effect measure(s) (e.g. risk ratio, mean difference) used in the synthesis or presentation of results. | Page 9 |
| Synthesis methods | 13a | Describe the processes used to decide which studies were eligible for each synthesis (e.g. tabulating the study intervention characteristics and comparing against the planned groups for each synthesis (item #5)). | Page 9 |
|  | 13b | Describe any methods required to prepare the data for presentation or synthesis, such as handling of missing summary statistics, or data conversions. | Page 9 |
|  | 13c | Describe any methods used to tabulate or visually display results of individual studies and syntheses. | Page 9 |
|  | 13d | Describe any methods used to synthesize results and provide a rationale for the choice(s). If meta-analysis was performed, describe the model(s), method(s) to identify the presence and extent of statistical heterogeneity, and software package(s) used. | Page 9 |
|  | 13e | Describe any methods used to explore possible causes of heterogeneity among study results (e.g. subgroup analysis, meta-regression). | Page 9 |
|  | 13f | Describe any sensitivity analyses conducted to assess robustness of the synthesized results. | Page 9 |
| Reporting bias | 14 | Describe any methods used to assess risk of bias due to missing results in a synthesis (arising from reporting biases). | Page 9 |

| **Section and Topic** | **Item #** | **Checklist item** | **Location where item**  **is reported** |
| --- | --- | --- | --- |
| assessment |  |  |  |
| Certainty assessment | 15 | Describe any methods used to assess certainty (or confidence) in the body of evidence for an outcome. | Page 9 |
| **RESULTS** | | |  |
| Study selection | 16a | Describe the results of the search and selection process, from the number of records identified in the search to the number of studies included in the review, ideally using a flow diagram. | Page 10-11 |
|  | 16b | Cite studies that might appear to meet the inclusion criteria, but which were excluded, and explain why they were excluded. | Page 11 |
| Study characteristics | 17 | Cite each included study and present its characteristics. | Page 11-16 |
| Risk of bias in studies | 18 | Present assessments of risk of bias for each included study. | Page 12-16 |
| Results of individual studies | 19 | For all outcomes, present, for each study: (a) summary statistics for each group (where appropriate) and (b) an effect estimate and its precision (e.g. confidence/credible interval), ideally using structured tables or plots. | Page 17-18 |
| Results of syntheses | 20a | For each synthesis, briefly summarise the characteristics and risk of bias among contributing studies. | Page 12-16 |
|  | 20b | Present results of all statistical syntheses conducted. If meta-analysis was done, present for each the summary estimate and its precision (e.g. confidence/credible interval) and measures of statistical heterogeneity. If comparing groups, describe the direction of the effect. | Page 17 |
|  | 20c | Present results of all investigations of possible causes of heterogeneity among study results. | Page 17 |
|  | 20d | Present results of all sensitivity analyses conducted to assess the robustness of the synthesized results. | Page 17 |
| Reporting biases | 21 | Present assessments of risk of bias due to missing results (arising from reporting biases) for each synthesis assessed. | Page 12-16 |
| Certainty of evidence | 22 | Present assessments of certainty (or confidence) in the body of evidence for each outcome assessed. | Page 17-21 |
| **DISCUSSION** | | |  |
| Discussion | 23a | Provide a general interpretation of the results in the context of other evidence. | Page 31-47 |
|  | 23b | Discuss any limitations of the evidence included in the review. | Page 45-46 |
|  | 23c | Discuss any limitations of the review processes used. | Page 45-46 |
|  | 23d | Discuss implications of the results for practice, policy, and future research. | Page 45-46 |
| **OTHER INFORMATION** | | |  |
| Registration and protocol | 24a | Provide registration information for the review, including register name and registration number, or state that the review was not registered. | Page 6 |
|  | 24b | Indicate where the review protocol can be accessed, or state that a protocol was not prepared. | Page 6 |
|  | 24c | Describe and explain any amendments to information provided at registration or in the protocol. | Page 6 |
| Support | 25 | Describe sources of financial or non-financial support for the review, and the role of the funders or sponsors in the review. | Page 47 |
| Competing interests | 26 | Declare any competing interests of review authors. | Page 65 |
| Availability of data, code and other materials | 27 | Report which of the following are publicly available and where they can be found: template data collection forms; data extracted from included studies; data used for all analyses; analytic code; any other materials used in the review | Page 65 |

# S2 File. Search Strategies Searched on December 2025

## MEDLINE (PubMed)

| **No.** | **Search** | **Results** |
| --- | --- | --- |
| #1 | "Immunological Non Response"[Title/Abstract] OR INR[Title/Abstract] OR "CD4+ T-cell deficiency"[Title/Abstract] OR "Discordant response of CD4+ T Cells"[Title/Abstract] OR "Discordant Immunological Response"[Title/Abstract] OR "Failure to achieve CD4 response"[Title/Abstract] OR "Immune reconstitution failure"[Title/Abstract] OR " Immunological nonresponse"[Title/Abstract] OR "Impaired CD cell recovery"[Title/Abstract] OR "inadequate immunological responder"[Title/Abstract] OR "Incomplete CD4+ T cell recovery"[Title/Abstract] OR "Incomplete Immune reconstitution"[Title/Abstract] OR "Insufficient immunological restoration"[Title/Abstract] OR "Low-level CD4 T-cell repopulation"[Title/Abstract] OR "Low CD4 T-cell reconstitution"[Title/Abstract] OR "Nonimmune reconstituters"[Title/Abstract] OR "Paradoxal Immunological response"[Title/Abstract] OR "Poor CD4+ T-cell recovery"[Title/Abstract] OR "Suboptimal CD4+ T-cell recovery"[Title/Abstract] OR "Suboptimal immune responders"[Title/Abstract] OR "Virologic-only response"[Title/Abstract] | 14,119 |
| #2 | "HIV"[MeSH Terms] | 112,889 |
| #3 | "Human Immunodeficiency Virus"[Title/Abstract] OR "HIV"[Title/Abstract] OR "Acquired Immunodeficiency Syndrome Virus"[Title/Abstract] OR "AIDS Virus"[Title/Abstract] OR "Human T-Lymphotropic Virus"[Title/Abstract] OR "HTLV"[Title/Abstract] | 432,132 |
| #4 | #2 OR #3 | 440,792 |
| #5 | "Anti-Retroviral Agents"[MeSH Terms] | 78,119 |
| #6 | "antiretroviral therapy, highly active"[MeSH Terms | 23,140 |
| #7 | "Antiretroviral Therapy"[Title/Abstract] OR "Anti-retroviral Therapy"[Title/Abstract] OR "Antiretroviral Treatment"[Title/Abstract] OR "Anti-retroviral Treatment"[Title/Abstract] OR "Highly Active Anti Retroviral Therapy"[Title/Abstract] OR "ART"[Title/Abstract] OR "ARV"[Title/Abstract] OR "HAART"[Title/Abstract] | 261,449 |
| #8 | #5 OR #6 OR #7 | 307,721 |
| #9 | **#1 AND #4 AND #8** | **272** |

## CENTRAL (Cochrane Library)

| **No.** | **Search** | **Results** |
| --- | --- | --- |
| #1 | ("Immunological Non Response" OR INR OR "CD4+ T-cell deficiency" OR "Discordant response of CD4+ T Cells" OR "Discordant Immunological Response" OR "Failure to achieve CD4 response" OR "Immune reconstitution failure" OR " Immunological nonresponse" OR "Impaired CD cell recovery" OR "inadequate immunological responder" OR "Incomplete CD4+ T cell recovery" OR "Incomplete Immune reconstitution" OR "Insufficient immunological restoration" OR "Low-level CD4 T-cell repopulation" OR "Low CD4 T-cell reconstitution" OR "Nonimmune reconstituters" OR "Paradoxal Immunological response" OR "Poor CD4+ T-cell recovery" OR "Suboptimal CD4+ T-cell recovery" OR "Suboptimal immune responders" OR "Virologic-only response"):ti,ab,kw | 3,472 |
| #2 | MeSH descriptor: [HIV] explode all trees | 4,257 |
| #3 | ("Human Immunodeficiency Virus" OR "HIV" OR "Acquired Immunodeficiency Syndrome Virus" OR "AIDS Virus" OR "Human T-Lymphotropic Virus" OR "HTLV"):ti,ab,kw | 33,388 |
| #4 | #2 OR #3 | 33,388 |
| **#5** | MeSH descriptor: [Anti-Retroviral Agents] explode all trees | 6,219 |
| #6 | MeSH descriptor: [Antiretroviral Therapy, Highly Active] explode all trees | 1,638 |
| #7 | ("Antiretroviral Therapy" OR "Anti-retroviral Therapy" OR "Antiretroviral Treatment" OR "Anti-retroviral Treatment" OR "Highly Active Anti Retroviral Therapy" OR "ART" OR "ARV" OR "HAART"):ti,ab,kw | 16,514 |
| #8 | #5 OR #6 OR #7 | 19,100 |
| **#9** | **#1 AND #4 AND #8** | **38** |

## Web of Science (WoS)

**TOPIC**: "Immunological Non Response" OR INR OR "CD4+ T-cell deficiency" OR "Discordant response of CD4+ T Cells" OR "Discordant Immunological Response" OR "Failure to achieve CD4 response" OR "Immune reconstitution failure" OR " Immunological nonresponse" OR "Impaired CD cell recovery" OR "inadequate immunological responder" OR "Incomplete CD4+ T cell recovery" OR "Incomplete Immune reconstitution" OR "Insufficient immunological restoration" OR "Low-level CD4 T-cell repopulation" OR "Low CD4 T-cell reconstitution" OR "Nonimmune reconstituters" OR "Paradoxal Immunological response" OR "Poor CD4+ T-cell recovery" OR "Suboptimal CD4+ T-cell recovery" OR "Suboptimal immune responders" OR "Virologic-only response"

**TOPIC:** "Human Immunodeficiency Virus" OR HIV OR "Acquired Immunodeficiency Syndrome Virus" OR "AIDS Virus" OR "Human T-Lymphotropic Virus" OR HTLV

**TOPIC:** "Antiretroviral Therapy" OR "Anti-retroviral Therapy" OR "Antiretroviral Treatment" OR "Anti-retroviral Treatment" OR "Highly Active Anti Retroviral Therapy" OR ART OR ARV OR HAART

**Results: 267**

## ScienceDirect

(“Immunological Non Response” OR “Discordant Immunological Response” OR “Suboptimal Immune Responders) AND (“Human Immunodeficiency Virus” OR HIV) AND (“Antiretroviral Therapy” OR “Antiretroviral Treatment” OR “Highly Active Antiretroviral Therapy” OR ART)

**Results: 19**

## ProQuest

“Immunological Non Response” AND (“Human Immunodeficiency Virus” OR HIV) AND (“Antiretroviral Therapy” OR “Antiretroviral Treatment” OR “Highly Active Antiretroviral Therapy”)

**Results: 44**

# S1 Fig. Odds ratio (OR) of demographical factor: being male (vs female) related to the risk of INR.

A

B

**Supplementary Figure S1**. Odds ratio (OR) of demographical factor: being male (vs female) related to the risk of INR. The pooled effect indicates that INR tends to be more significantly frequent in the male population [OR 1.33, 95% CI (1.12-1.58), P = 0.0013, I2 = 66,7%]. Egger Test result: t = 1.70, df = 30, p-value = 0.0998, Bias estimate: 0.8757 (SE = 0.5156). (A) Forest plot, (B) Funnel plot. CI, confidence interval; SE, standard error.

# S2 Fig. Mean difference (MD) of demographical factor: age-related to the risk of INR

A

B

**Supplementary Figure S2**. Mean difference (MD) of demographical factor: age-related to the risk of INR. The pooled effect indicates that INR tends to be more significantly frequent in the older population [MD 2.61, 95% CI (1.42-3.81), P = < 0.0001, I2 = 97,6%]. Egger Test result: t = -2.03, df = 24, p-value = 0.0534, Bias estimate: -2.8308 (SE = 1.3931). (A) Forest plot, (B) Funnel plot. CI, confidence interval; SE, standard error.

# S3 Fig. Odds ratio (OR) of demographical factor: transmission route related to the risk of INR

A

B

**Supplementary Figure S3**. Odds ratio (OR) of demographical factor: transmission route related to the risk of INR. The pooled effect indicates the significance in intravenous drug use in the INR group [OR 1.67, 95% CI (1.15-2.42), P = 0.0074, I2 = 61,2%]. However, there is no significant difference regarding the heterosexual transmission route in both groups [OR 0.97, 95% CI (0.71-1.33), P = = 0.8379, I2 = 85,7%] and homosexual transmission route in the IR group [OR 0.64, 95% CI (0.52-0.80), P < 0.0001, I2 = 63,9%]. Egger Test result: t = 0.59, df = 45, p-value = 0.5614, Bias estimate: 0.3952 (SE = 0.6755). (A) Forest plot, (B) Funnel plot. CI, confidence interval; SE, standard error.

# S4 Fig. Odds ratio (OR) of demographical factor: having lower education (vs. higher education) related to the risk of INR

**Supplementary Figure S4.** Odds ratio (OR) of demographical factor: having lower education (vs. higher education) related to the risk of INR. The forest plot shows there is no significant difference regarding INR events between higher and lower education groups. The pooled estimate presented a higher risk of INR condition in the lower education group [OR 1.15, 95% CI (0.88-1.50), P = 0.3038, I2 = 77%]. CI, confidence interval.

# S5 Fig. Odds ratio (OR) of clinical factor: coinfection related to the risk to INR

A

B

**Supplementary Figure S5**. Odds ratio (OR) of clinical factor: coinfection related to the risk to INR. The pooled effect indicates significant coinfection rates, including hepatitis A, B, C, and tuberculosis in patients with INR condition [OR = 1.58, 95% CI (1.41-1.77), P < 0.0001, I2 = 37,5%]. For the individual subgroup, there are significant differences in hepatitis B coinfection [OR = 1.30, 95% CI (1.08-1.57), P = 0.0061, I2 = 24,1%], hepatitis C coinfection [OR = 1.85, 95% CI (1.50-2.28), P < 0.0001, I2 = 17,9%] and tuberculosis coinfection [OR = 1.77, 95% CI (1.41-2.21), P < 0.0001, I2 = 63,8%] with the higher rates in the INR group. However, only one study reported the hepatitis A coinfection case, so there will be no effect estimated. Egger Test result: t = -0.58, df = 35, p-value = 0.5688, Bias estimate: -0.2360 (SE = 0.4103). (A) Forest plot, (B) Funnel plot. CI, confidence interval; SE, standard error.

# S6 Fig. Odds ratio (OR) of clinical factor: body mass index underweights (vs. normal) related to the INR

**Supplementary Figure S6**. Odds ratio (OR) of clinical factor: body mass index underweights (vs. normal) related to the INR. The forest plot presents is no significant difference regarding in INR event between the underweight and normal weight groups. The pooled estimate presented a higher risk of INR condition in the underweight group [OR 1.30, 95% CI (0.81-2.10), P = 0.2766, I2 = 88,9%]. CI, confidence interval.

# S7 Fig. Odds ratio (OR) of clinical factor: WHO HIV stage III-IV (vs. stage I-II) related to the risk of INR

**Supplementary Figure S7**. Odds ratio (OR) of clinical factor: WHO HIV stage III-IV (vs. stage I-II) related to the risk of INR. The forest plot shows a significant difference in INR events between the stage I-II and stage III-IV groups. The pooled estimate presented a higher stage of HIV is related to the INR event [OR 2.66, 95% CI (2.34-3.02), P < 0.0001, I2 = 80,6%]. CI, confidence interval.

# S8 Fig. Standardized Mean Difference (SMD) of clinical factor: baseline CD4+ T Cells count related to the risk of INR

**Supplementary Figure S8**. Standardized Mean Difference (SMD) of clinical factor: baseline CD4+ T Cells count related to the risk of INR. The forest plot indicates that INR tends to be more significantly frequent in the PLHIV with lower baseline CD4+ T Cells Count [SMD -3.82, 95% CI (-4.94-(-2.70)), P < 0.0001, I2 = 99,3%].

# S9 Fig. Standardized Mean Difference (SMD) of clinical factor: Nadir CD4+ T Cells count related to the risk of INR

**Supplementary Figure S9**. Standardized Mean Difference (SMD) of clinical factor: Nadir CD4+ T Cells count related to the risk of INR. The forest plot indicates that INR tends to be more significantly frequent in the PLHIV with lower nadir CD4+ T Cells Count [SMD -1.69, 95% CI ( -2.69-(-0.68)), P = 0.0010, I2 = 99,1%].

# S1 Table. Newcastle-Ottawa Scale (NOS) Quality assessment of the included cohort studies

| **Author** | **Selection** | | | | **Comparability of cohorts on the basis of the design or analysis** | **Exposure** | | | **Study Quality** | |
| --- | --- | --- | --- | --- | --- | --- | --- | --- | --- | --- |
|  | **Representativeness of the exposed cohort** | **Selection of the non-exposed cohort** | **Ascertainment of exposure** | **Demonstration that outcome of interest was not present at start of**  **study** |  | **Assessment of outcome** | **Enough follow-up time length for outcome to occur** | **Adequacy of follow-up of cohorts** | **Total score** | **Judgment** |
| *Zhao et al., 2024* [1] | * | * | * | * | ** | * | * | * | 9 | Good |
| *Vos et al., 2024* [2] | * | * | * | * | ** | * | * | No Star | 8 | Good |
| *Zhang et al., 2023* [3] | * | * | * | * | ** | * | * | * | 9 | Good |
| *Wen et al., 2023* [4] | * | * | * | * | ** | * | * | * | 9 | Good |
| *Taramasso et al., 2023* [5] | * | * | * | * | ** | * | * | * | 9 | Good |
| *Kacker et al., 2023* [6] | * | * | * | * | * | * | * | * | 8 | Good |
| *Pereira et al., 2022* [7] | * | * | * | * | ** | * | * | * | 9 | Good |
| *Handoko et al., 2020* [8] | * | * | * | * | ** | * | * | * | 9 | Good |
| *Li et al.,*  2019 [9] | * | * | * | * | ** | * | * | * | 9 | Good |
| *Engsig et al., 2010* [10] | * | * | * | * | ** | * | * | * | 9 | Good |
| *Nicastri et al., 2005* [11] | * | * | * | No Star | * | * | * | No Star | 6 | Moderate |
| *Kaufmann et al., 2005* [12] | * | * | * | No Star | * | * | * | No Star | 6 | Moderate |
| *Sembiring et al., 2024* [13] | * | * | * | * | * | * | No Star | No Star | 6 | Moderate |
| *Guedes et al.,*  2023 [14] | * | * | * | * | * | * | * | No Star | 7 | Good |
| *Fan et al., 2023* [15] | * | * | * | * | ** | * | * | * | 9 | Good |
| *Carvalho-Silva et al.,* 2020 [16] | * | No Star | * | * | * | * | No Star | No Star | 5 | Moderate |
| *Jiang et al.,* 2018 [17] | No Star | * | No Star | * | * | * | * | No Star | 5 | Moderate |
| *Moore et al.,*  2007 [18] | * | No Star | * | * | ** | * | * | * | 8 | Good |
| *Chen et al.,* 2022 [19] | * | No Star | * | * | ** | * | * | * | 8 | Good |
| *Bayarsaikhan et al.,* 2021 [20] | * | No Star | * | * | * | * | * | * | 7 | Good |

| *Li et al.,* 2019 [21] | * | * | * | * | ** | * | * | * | 9 | Good |
| --- | --- | --- | --- | --- | --- | --- | --- | --- | --- | --- |
| *Boatman et al.,*  2019 [22] | * | * | * | * | ** | * | * | * | 9 | Good |
| *Han et al.,* 2020 [23] | * | * | * | * | ** | * | * | * | 9 | Good |
| *He et al.,* 2016 [24] | * | * | * | * | ** | * | * | * | 9 | Good |
| *Lembas et al.,*  2022 [25] | * | * | * | * | * | * | * | * | 8 | Good |
| *Rb-Silva et al.,*  2019 [26] | * | * | * | * | * | * | * | * | 8 | Good |
| *Greenblatt et al.,*  2019 [27] | * | * | * | * | * | * | * | * | 8 | Good |
| *Bahemana et al.,*  2020 [28] | * | * | * | * | * | * | * | * | 8 | Good |
| *Gunda et al.,*  2017 [29] | * | * | * | * | * | * | * | * | 8 | Good |
| *Fiseha et al.,*  2022 [30] | * | * | * | * | ** | * | * | * | 9 | Good |
| *Lei et al.,* 2022 [31] | * | * | * | * | ** | * | * | * | 9 | Good |
| *Noiman et al.,*  2022 [32] | * | * | * | * | ** | * | * | * | 9 | Good |
| *Tian et al., 2023*  [33] | * | * | * | * | No Star | * | * | * | 7 | Good |
| *Merlini et al., 2021* [34] | * | * | * | * | * | * | * | * | 8 | Good |
| *Shi, Y et al., 2025* [1] | * | * | * | * | * | * | * | * | 8 | Good |
| *Rachman, B.E et al., 2025 [2]* | * | * | * | * | ** | * | * | * | 9 | Good |
| *Sun, R et al., 2025 [3]* | * | * | * | * | ** | * | * | * | 9 | Good |

# S2 Table. Newcastle-Ottawa Scale (NOS) Quality assessment of the included case control studies.

| **Author** | **Selection** | | | | **Comparability of cases and controls on the basis of the design or analysis** | **Exposure** | | | **Study Quality** | |
| --- | --- | --- | --- | --- | --- | --- | --- | --- | --- | --- |
|  | **Adequateness of the case definition** | **Representativeness of the cases** | **Selection of controls** | **Definition of controls** |  | **Ascertainment of exposure** | **Same method of**  **ascertainment for cases and controls** | **Non-**  **response rate** | **Total score** | **Judgment** |
| *Garcia et al.,*  2019 [35] | * | * | * | * | ** | * | * | No Star | 8 | Good |
| *Yeregui et al.,*  2020 [36] | * | * | * | * | ** | * | * | No Star | 8 | Good |
| *Coelho et al.,*  2018 [37] | * | No Star | * | * | ** | * | * | * | 8 | Good |
| *Qian et al.,*  2021 [38] | * | * | * | No Star | * | * | * | * | 7 | Good |
| *Younes et al.,*  2018 [39] | * | * | No Star | * | ** | * | * | * | 8 | Good |
| *Yeregui et al.,*  2022 [40] | * | * | No Star | * | ** | * | * | * | 8 | Good |
| *El-Beeli et al.,*  2016 [41] | * | * | No Star | * | ** | * | * | * | 8 | Good |
| *Soria et al.,*  2011 [42] | * | * | No Star | * | ** | * | * | * | 8 | Good |
| *Restrepo et al.,*  2019 [43] | * | * | No Star | * | ** | * | * | * | 8 | Good |
| *Teshome et al.,*  2014 [44] | * | * | * | * | * | * | * | No Star | 7 | Good |

# S3 Table. Newcastle-Ottawa Scale (NOS) Quality assessment of the included cross-sectional studies

| **Author** | **Selection** | | | | **Comparability of subjects in different outcome groups on the basis of design or analysis** | **Exposure** | | **Study Quality** | |
| --- | --- | --- | --- | --- | --- | --- | --- | --- | --- |
|  | **Representativeness of the sample** | **Sample size** | **Non-respondents** | **Ascertainment of exposure** |  | **Assessment of outcome** | **Statistical test** | **Total score** | **Judgment** |
| *Lao et al.,*  2022 [45] | * | * | * | * | ** | * | * | 8 | Good |
| *Nabatanzi et al.,*  2023 [46] | No Star | No Star | * | ** | * | ** | * | 8 | Good |
| *Nguyen et al.,*  2016 [47] | No Star | * | * | * | ** | ** | * | 8 | Good |
| *Masson et al., 2018* [48] | No Star | No Star | No Star | ** | ** | ** | * | 7 | Good |
| *Gelba et al.,*  2020 [49] | * | * | * | * | ** | ** | * | 9 | Good |
| *Sun et al.,*  2017 [50] | * | * | * | * | ** | ** | * | 9 | Good |
| *Ferrari,*  *2022* [51] | No Star | * | * | * | ** | ** | * | 8 | Good |
| Tang *et al*.,  2024 [4] | * | no star | no star | * | no star | * | * | 4 | Moderate |
| Muñoz-Muela *et al*., 2024 [5] | * | * | * | no star | ** | * | no star | 6 | Moderate |
| Tian *et al*.,  2025 [6] | * | * | * | no star | ** | * | * | 7 | Good |
| Rins-Lozano *et al*., 2025 [7] | * | * | * | no star | no star | * | * | 5 | moderate |
| Tang *et al*.,  2024 [4] | * | * | * | no star | ** | * | no star | 6 | moderate |

# S4 Table. Exploratory meta-regression of study-level ICU, HCV, and HBV prevalence on the association between sex and INR

| **Moderator** | **k** | **β per +10%** | **95% CI** | **p** | **exp(β)** | **R²** |
| --- | --- | --- | --- | --- | --- | --- |
| IDU | 11 | −0.1097 | −0.1786 to −0.0407 | 0.0058 | 0.896 | 90.0% |
| HCV | 14 | −0.1078 | −0.2710 to 0.0554 | 0.176 | 0.898 | 16.7% |
| HBV | 13 | +0.1140 | −0.5007 to 0.7287 | 0.691 | 1.121 | 0% |
